# Evaluating the strengths of salt bridges in the CutA1 protein using molecular dynamic simulations: a comparison of different force fields

**DOI:** 10.1002/2211-5463.12731

**Published:** 2019-09-27

**Authors:** Yoshinori Matsuura, Yasumasa Joti, Bagautdin Bagautdinov, Katsuhide Yutani

**Affiliations:** ^1^ RIKEN SPring‐8 Center Sayo Hyogo Japan; ^2^ Japan Synchrotron Radiation Research Institute Sayo Hyogo Japan; ^3^ Present address: Technical Department Tottori University Tottori Japan

**Keywords:** charged residue, conformational stability of proteins, CutA1 protein, electrostatic energy, MD simulation, salt bridge

## Abstract

Ion–ion interactions (salt bridges) between favorable pairs of charged residues are important for the conformational stability of proteins. Molecular dynamic (MD) simulations are useful for elucidating the interactions among charged residues fluctuating in solution. However, the quality of MD results depends strongly on the force fields used. In this study, we compared the strengths of salt bridges among force fields by performing MD simulations using the CutA1 protein (trimer) from the hyperthermophile *Pyrococcus horikoshii* (PhCutA1), which has an unusually large proportion of charged residues. The force fields Chemistry at HARvard Macromolecular Mechanics (Charmm)27, Assisted Model Building and Energy Refinement (Amber)99sb, Amber14sb, GROningen Molecular Simulation (Gromos)43a1, and Gromos53a6 were used in combination with two different water models, tip3p (for Charmm27, Amber99sb, and Amber14sb) and simple point charge/extended (for Amber99sb, Gromos43a1, and Gromos53a6), yielding a total of six combinations. The RMSDs of all Cα atoms of PhCutA1 were similar among force fields, except for Charmm27, during 400‐ns MD simulations at 300 K; however, the radius of gyration (*R*_g_) was greater for Amber99sb and shorter for Gromos43a1. The average strengths of salt bridges for each positively charged residue did not differ greatly among force fields, but the strengths at specific sites within the structure depended sensitively on the force field used. In the case of the Gromos group, positively charged residues could engage in favorable interactions with many more charged residues than in the other force fields, especially in loop regions; consequently, the apparent strength at each site was lower.

**Abbreviations**
Amber: Assisted Model Building and Energy Refinement
Charmm: Chemistry at HARvard Macromolecular Mechanics
GROMACS: GROningen MAchine for Chemical Simulations
Gromos: GROningen Molecular Simulation
MD: molecular dynamics
RMSF: root‐mean‐square fluctuation
spc/e: simple point charge/extended
tip3p: transferable intermolecular potential 3P

The proteins of hyperthermophiles, which grow at temperatures exceeding 80 °C, have higher proportions of charged residues than those of mesophiles 1, 2. These proteins are thought to be stabilized by ion–ion interactions among charged residues 2, 3, 4, 5, 6, 7, 8, 9, 10, 11, 12, 13, 14, 15. Structural evidence for these interactions has mainly been obtained from X‐ray crystal structures 16, 17, 18, 19. Many charged residues are located on the surface of protein molecules; however, the solution structures of surface residues are often poorly reflected by crystallographic data, largely due to artifacts of crystallization and cooling to 100 K. Furthermore, in X‐ray snapshots residues visualized are fixed, even though in solution they might fluctuate; this is particularly true of charged residues on the protein surface. Molecular dynamics (MD) simulations can be used to elucidate the structural features of these residues in solution 20, 21, 22, 23, 24, 25, 26. However, because the quality of MD results depends strongly on the force fields used 27, it is important to understand how the strengths of ion–ion interactions (salt bridges) are influenced by different force fields.

The CutA1 protein from hyperthermophile *Pyrococcus horikoshii* (PhCutA1) has a high denaturation temperature (*T*_d_) (nearly 150 °C) and an unusually high percentage of charged residues [42.2% vs. 22.3% for CutA1 from *Escherichia coli* (EcCutA1)] 16. The *T*_d_ of a hydrophobic and ionic mutant of EcCutA1 (with a *T*_d_ of nearly 90 °C) has been successfully improved to 142 °C, near the *T*_d_ of PhCutA1 15. Many mutants of PhCutA1 in which the charged residues were replaced by noncharged residues have been examined 17. The X‐ray crystal structure of PhCutA1 reveals that the protein exists as a tightly intertwined trimer that clearly resembles EcCutA1. Each trimer contains three identical inter‐subunit interfaces, in which both edges of one strand (β‐2) interact with the edges of the β‐2 strand in the other two subunits (Fig. 1). Charged residues in PhCutA1 engage in many intra‐ and inter‐subunit interactions, whose stabilities have been extensively examined in mutation studies 17. Close examination of these residues might provide insight into how the strengths of salt bridges change in MD simulations using different force fields.

**Figure 1:**
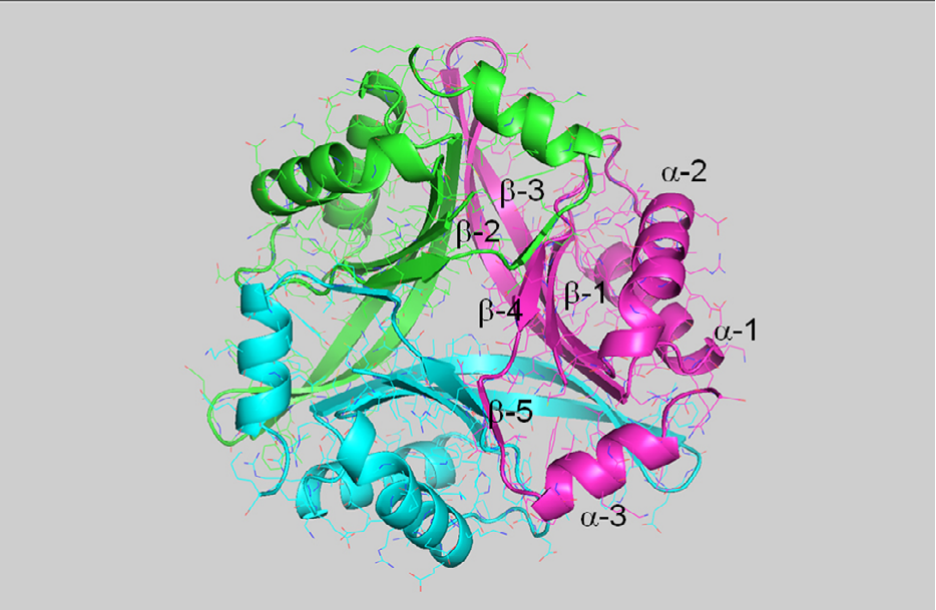
The trimer structure of PhCutA1 (A, B, and C subunits of PDB ID 4nyo). Different colors represent different chains. α and β represent α‐helix and β‐strand, respectively.

In this study, we used the trimer structure of PhCutA1 (102 × 3 residues) to compare the strengths of salt bridges in simulations using various force fields (Table 1). GROningen Molecular Simulation (Gromos)43a1 28 and Gromos53a6 29 are united‐atom representations for aliphatic CH_n_ groups, whereas Chemistry at HARvard Macromolecular Mechanics (Charmm)27 30, Assisted Model Building and Energy Refinement (Amber)99sb 31, and Amber14sb 32 are all‐atom representations. For water models, we used transferable intermolecular potential 3P (tip3p) 33 and simple point charge/extended (spc/e) 34. Charmm27, Amber99sb, and Amber14sb were used with tip3p, and Amber99sb, Gromos43a1, and Gromos53a6 were used with spc/e, yielding a total of six combinations (hereafter, referred to simply as ‘the six force fields’). We performed 400‐ns MD simulations of PhCutA1 (trimer) at 300 K using these six force fields (Table 1) and analyzed the influence of the various force fields on the strengths of salt bridges on the basis of the locations of charged residues in the PhCutA1 X‐ray structure.

**Table 1:** List of six force fields used in MD simulations, and comparison of RMSD, *R*_g_, and RMSF for each force field (50‐400 ns).

	Force fields	Water model	RMSD (nm)	*R*_g_ (nm)	RMSF (nm)
1	Charmm27	tip3p	0.09 ± 0.01	1.93 ± 0.01	0.06 ± 0.02
2	Amber99sb	spc/e	0.17 ± 0.01	1.99 ± 0.01	0.08 ± 0.03
3	Amber99sb	tip3p	0.17 ± 0.02	1.98 ± 0.01	0.08 ± 0.03
4	Amber14sb	tip3p	0.17 ± 0.02	1.94 ± 0.01	0.06 ± 0.02
5	Gromos43a1	spc/e	0.17 ± 0.01	1.90 ± 0.01	0.08 ± 0.04
6	Gromos53a6	spc/e	0.17 ± 0.02	1.95 ± 0.01	0.08 ± 0.04

## Results and Discussion

### Differences in general characteristics of PhCutA1 among MD simulations using six force fields

**Figure 2:**
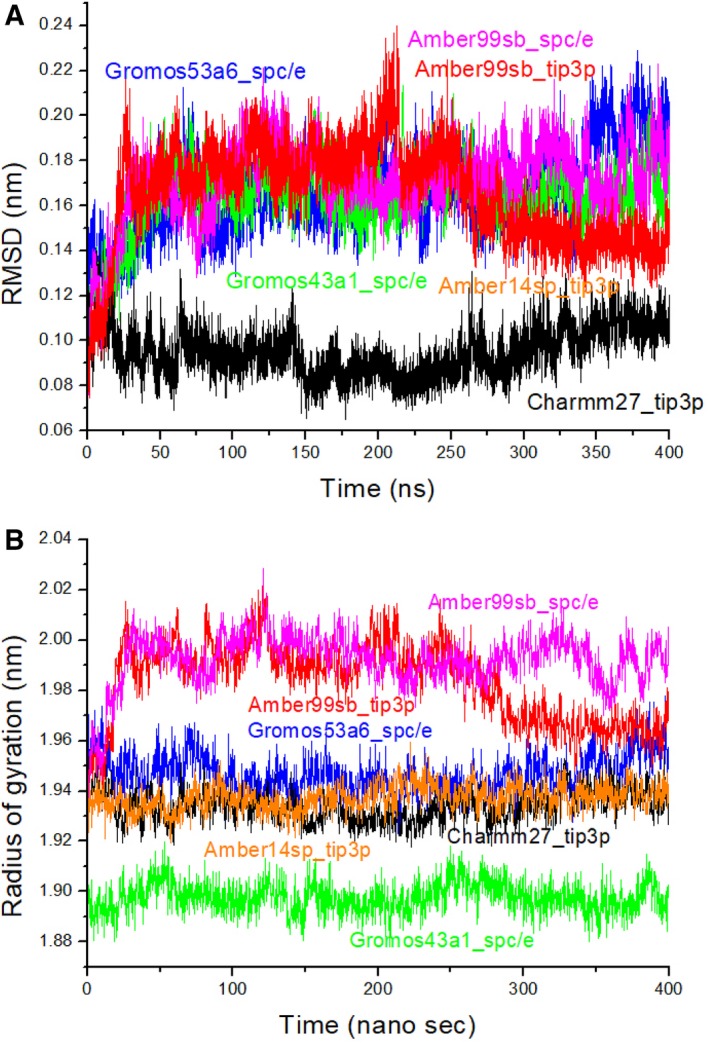
Trajectories of RMSD of Cα atoms (A) and *R*_g_ (B) for PhCutA1 at six different force fields over 400‐ns of MD simulations. Black, magenta, red, orange, green, and blue represent Charmm27_tip3p, Amber99sb_spc/e, Amber99sb_tip3p, Amber14sb_tip3p, Gromos43a1_spc/e, and Gromos53a6_spc/e, respectively.

Figure 2A shows the trajectories of the RMSDs of all C_⍺_ atoms of PhCutA1 in MD simulations performed at 300 K using six different force fields. As shown in the figure, RMSDs were similar after 50 ns, except in the case of Charmm27_tip3p, for which the value was smaller RMSD (Table 1). On the other hand, the radius of gyration (*R*_g_) of PhCutA1 differed significantly among the six force fields (Fig. 2B): lowest for Gromos43a1_spc/e (a compact structure) and highest for Amber99sb_spc/e and Amber99sb_tip3p (a loose structure) (Table 1). The largest difference in *R*_g_ was 0.096 nm, about 5% of the highest value. This large difference between force fields was also observed in a mutant EcCutA1 in an MD simulation at 300 K (in press). The difference in *R*_g_ among force fields might affect the strengths of salt bridges between the subunits of PhCutA1.

The average residue numbers of secondary structures in PhCutA1 in MD simulations at 300 K using each of the six force fields are listed in Table S2. The MD‐simulated PhCutA1 proteins exhibited similar secondary structures, including β‐sheet, ⍺‐helix, β‐bridge, and turn. The numbers of residues in β‐sheet and α‐helix structures were similar among the six force fields, although there were some slight differences. The difference in helicity at each residue of PhCutA1 was examined in 50–400‐ns MD simulations at 300 K using the six force fields (Fig. S1). In the regions of the ⍺‐1, ⍺‐2, and ⍺‐3 helices, Charmm27_tip3p yielded relatively high helicity, as shown in the figure, suggesting that the α‐helical structure of Charmm27_tip3p is more stable in a long region of α‐helix than those of the other force fields, although the average number of residues in α‐helix structure was not the highest for Charmm27_tip3p (Table S2). On the other hand, the α‐helical structure of Amber99sb_tip3p seemed to be unstable in all three α‐helix regions. Yoda *et al*. 35 reported that for small peptides, the Amber force field favors ⍺‐helix, whereas Gromos favors β‐hairpin.

The average root‐mean‐square fluctuations (RMSF) of the C_⍺_ atoms of PhCutA1 from 50 to 400‐ns MD simulations at 300 K using the six force fields are shown in Fig. S2A. The differences in average RMSF of each Cα atom were obtained by subtracting the average values of Cα atoms from the value for each Cα atom (Fig. S2B). In the N‐terminal region, the fluctuations of Amber99sb_spc/e and Amber99sb_tip3p were larger than those in the other force fields, whereas in the C‐terminal region, the fluctuations of Gromos43a1_spc/e and Gromos53a6_spc/e were larger. The loop region between positions 41 and 45 fluctuated considerably in all cases, but the fluctuations were larger in the three used with the spc/e water model, and smaller in the cases of Amber99sb_tip3p and Amber14sb_tip3p. In the loop regions near positions 75 and 90, fluctuations were larger in the case of Amber99sb_tip3p than the other force fields. The average values of the fluctuations in Charmm27_tip3p and Amber14sb_tip3p were smaller than those of the other force fields (Table 1).

### Evaluation of the strengths of salt bridges in PhCutA1 during MD simulations at 300 K

In MD simulations, the strengths of salt bridges in proteins differ depending on the force fields used 36, 37, 38. Hence, we compared the formation of salt bridges in PhCutA1 in 300 K MD simulations using six different force fields. Specifically, we examined favorable intra‐subunit interactions of 186 residues and favorable inter‐subunit interactions of 60 residues with Arg or Lys in the trimer of PhCutA1 (Table S1A, B). Interactions were selected when the distance between favorable pairs of charged residues was < 0.6 nm at least once among 31 structural snapshots obtained from MD simulations of PhCutA1 using Gromos43a1_spc/e; the snapshots were acquired every 10 ns for 400 ns after an initial 100‐ns run.

**Figure 3:**
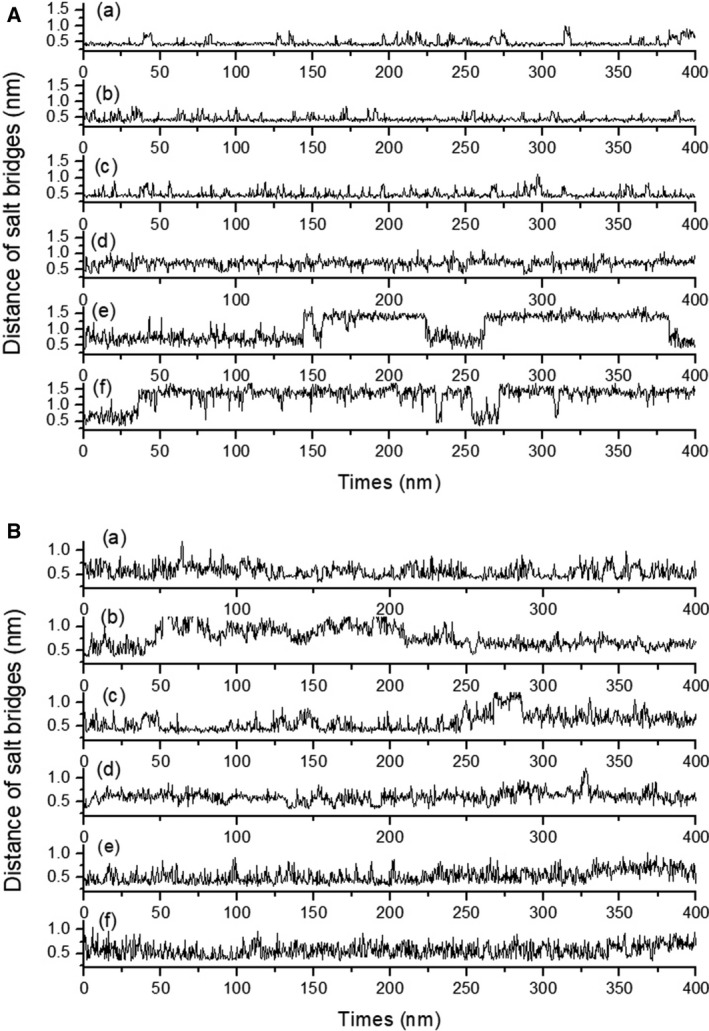
Trajectory of the length of typical salt bridges in PhCutA1 at different force fields. (a), (b), (c), (d), (e), and (f) represent Charmm27_tip3p, Amber99sb_spc/e, Amber99sb_tip3p, Amber14sb_tip3p, Gromos43a1_spc/e, and Gromos53a6_spc/e, respectively. (A)The distances between the C_ε_ atom of Lys101 and the C_δ_ atom of Glu64 in the A subunit. (B) The distances between the C_ε_ atom of Lys70 in the C subunit and the C_γ_ atom of Asp91 in the A subunit.

Figure 3A,B shows typical trajectories of the distances of intra‐ and inter‐subunits, respectively, during 400‐ns MD simulations at 300 K. Figure 3A represents the distance between the C_ε_ atom of Lys101 and the C_δ_ atom of Glu64 in the A subunit of PhCutA1 during MD simulations using all six force fields. In the cases of Charmm27_tip3p and Amber99sb_spc/e, large fluctuations were not detected, and the lengths of salt bridges between Lys101 and Glu64 were 0.47 ± 0.10 nm and 0.46 ± 0.10 nm, respectively (Table S3A). The trajectories of Gromos43a1_spc/e and Gromos53a6_spc/e fluctuated considerably; the lengths of ion–ion interactions were 1.20 ± 0.34 nm and 1.27 ± 0.32 nm, respectively. On the other hand, Fig. 3B shows the trajectory of the distance between the C_ε_ atom of Lys70 in the C subunit of PhCutA1 and the C_γ_ atom of Asp91 in the A subunit during MD simulations using the six force fields. The inter‐subunit interactions of Amber99sb_spc/e fluctuated the most, and the length of the ion–ion interaction was 0.78 ± 0.22 nm. By contrast, that of Gromos43a1_spc/e was 0.53 ± 0.14 (Table S3B), indicating formation of a stable salt bridge between subunits.

A salt bridge is considered to form when the distance between favorable pairs of charged residues is < 0.6 nm 22. We examined the percent occupancies of ion pairs with lengths below 0.6 nm for targeted intra‐ and interinteractions of 246 residues [186 residues for intra‐ (Table S1A) and 60 ones for inter‐subunit interactions (Table S1B)] of PhCutA1, as shown in Fig. S3. In this analysis, 100% occupancy indicates that all lengths of a favorable ion pair were < 0.6 nm during a 400‐ns MD simulation at 300 K. For example, as shown in Fig. S3A, Arg68 was expected to interact with five favorable residues (Glu24, Glu64, Glu67, Glu71, and C‐terminal) within the same subunit; however, in simulations using Charmm27_tip3p, Amber99sb_spc/e, and Amber99sb_tip3p, Arg68 interacted with Glu24 with almost 100% occupancy, but barely interacted with Glu64, Glu67, or the C terminus. The three bars in the figure depicting the interaction of Arg68 and Glu24 represent data from the A, B, and C subunits of PhCutA1. Figure S3 also shows the distance of salt bridges obtained from the crystal structure of PhCutA1.

### Difference in the strengths of salt bridges at specific sites in PhCutA1 during MD simulations using the six force fields

Table 2 shows percent occupancies of intra‐subunit salt bridges of PhCutA1 during 400‐ns MD simulations at 300 K. The average percent occupancy of intra‐subunit salt bridges for each positively charged residue ranged from 74.0% to 83.6% among the six force fields (Table 2A), and the average value was 79.4 ± 2.5%. For inter‐subunit salt bridges, the value ranged from 23.7% to 38.0% (Table 2B) with a mean of 30.3 ± 4.3%. These results indicate that the average strength of salt bridges for each positively charged residue of PhCutA1 was similar among the six force fields examined, within experimental error, and that the difference in force fields barely affected the average strength of salt bridges. However, the strengths of salt bridges at specific sites in the structure were significantly affected by the force field used, as described below.

**Table 2:** (A) Percent occupancies of intra‐subunit salt bridges in PhCutA1 during 400‐ns MD simulations at 300 K using the indicated force fields. (B) Percent occupancies of inter‐subunit salt bridges in PhCutA1 during 400‐ns MD simulations at 300 K using the indicated force fields. Data show average values of three subunits.

Targeted residues		Force fields					
Positively charged Residues	Interacting residues	Charmm27 _tip3p	Amber99sb _spc/e	Amber99sb _tip3p	Amber14sb _tip3p	Gromos43a1 _spc/e	Gromos53a6 _spc/e
(A)							
N‐terminal	Glu59	97.7	84.3	61.0	95.0	98.4	98.9
	Asp84	13.5	44.9	41.7	2.7	37.6	15.1
	Asp86	0.7	0.0	0.0	0.0	1.3	13.4
	Sum	111.8	129.2	102.7	97.7	137.3	127.3
Lys16	Asp10	0.0	0.1	0.0	0.0	0.0	0.0
	Glu12	28.6	29.4	32.9	40.3	54.2	47.8
	Glu15	2.0	1.2	2.1	1.3	0.9	0.8
	Sum	30.6	30.7	35.0	41.6	55.1	48.6
Lys19	Glu12	0.0	0.0	0.0	0.0	0.0	0.0
	Glu15	90.4	94.0	95.4	91.8	90.1	90.1
	Sum	90.4	94.0	95.4	91.8	90.1	90.1
Lys23	Glu24	4.3	1.2	1.4	7.5	6.4	8.3
Arg25	Glu24	0.0	0.0	0.0	0.0	0.3	0.0
	Glu98	0.0	0.1	0.0	0.1	0.4	0.0
	Glu99	97.2	96.1	91.3	99.3	86.5	94.9
	C‐terminal	0.0	0.0	0.0	0.0	2.9	1.1
	Sum	97.3	96.2	91.3	99.4	90.1	95.9
Arg33	Glu34	0.0	0.9	0.2	0.0	54.8	35.3
Arg36	Glu34	0.0	0.3	0.1	0.1	0.3	0.1
	Glu46	0.0	0.0	0.0	0.0	0.0	0.0
	Glu47	89.9	74.9	79.5	56.7	65.8	71.1
	Asp48	8.8	5.6	2.8	2.3	1.2	0.2
	Sum	98.7	80.8	82.4	59.0	67.4	71.3
Lys44	Glu42	19.6	12.2	14.4	8.3	5.6	1.8
	Glu46	64.9	49.8	40.4	32.2	41.0	60.4
	Glu47	0.0	0.0	0.0	0.1	1.8	0.5
	Sum	84.5	62.0	54.7	40.6	48.4	62.7
Lys49	Asp10	5.0	4.3	6.0	0.4	7.8	0.8
	Glu12	0.0	0.0	0.0	0.0	0.0	0.0
	Glu34	82.6	92.2	84.3	56.9	50.5	63.4
	Glu47	0.6	0.1	0.1	0.5	8.9	2.8
	Asp48	1.9	13.4	5.3	2.5	8.0	2.4
	Sum	90.1	110.0	95.8	60.2	75.2	69.3
Arg58	Glu59	0.2	0.0	0.3	0.2	5.4	1.4
	Asp60	99.9	98.5	99.8	99.8	79.5	93.0
	Glu98	0.0	0.0	0.0	0.0	0.8	0.0
	C‐terminal	32.4	35.9	30.6	47.0	16.7	17.1
	Sum	132.5	134.4	130.6	147.0	102.4	111.5
Lys66	Glu63	52.3	60.0	72.2	89.0	71.2	44.3
	Glu67	7.5	24.1	9.6	8.2	19.3	11.0
	Sum	59.8	84.1	81.8	97.2	90.5	55.2
Arg68	Glu24	99.5	99.5	95.6	53.7	58.2	63.2
	Glu64	9.0	3.0	3.5	37.4	24.4	19.6
	Glu67	1.8	0.7	3.5	45.3	14.5	6.4
	Glu71	83.6	92.2	93.0	72.1	46.4	27.8
	C‐terminal	0.0	0.0	0.0	0.0	0.2	0.0
	Sum	193.9	195.5	195.6	208.5	143.7	117.0
Lys70	Glu63	0.0	0.0	0.0	0.0	0.0	0.0
	Glu67	29.8	54.5	46.3	40.1	50.0	26.5
	Glu71	2.7	1.0	1.9	3.0	4.3	2.2
	Asp76	0.3	18.1	15.9	0.5	0.9	0.4
	Sum	32.8	73.6	64.1	43.6	55.2	29.1
Arg82	Glu59	27.9	46.0	50.0	45.7	51.7	58.9
	Asp60	0.0	0.0	0.0	0.0	0.0	0.0
	Glu63	0.0	0.0	0.1	0.0	0.1	0.0
	Asp84	99.6	2.3	1.1	80.8	6.7	86.8
	Sum	127.5	48.4	51.1	126.5	58.6	145.8
Lys94	Glu90	27.2	43.2	43.5	20.5	31.7	16.7
	Asp91	26.0	28.9	41.7	25.5	35.1	21.1
	Glu98	7.4	10.5	4.1	7.4	10.8	15.8
	Glu99	0.0	0.0	0.0	0.0	0.3	0.0
	Sum	60.7	82.7	89.3	53.4	78.0	53.6
Lys101	Glu24	10.8	10.4	12.6	15.6	3.7	4.2
	Asp60	0.0	0.0	0.0	0.0	4.3	1.2
	Glu64	86.8	88.0	80.2	14.5	7.7	6.5
	Glu98	0.0	0.0	0.0	0.0	32.9	10.1
	Glu99	0.0	0.0	0.0	0.0	4.0	6.9
	C‐terminal	38.7	52.0	46.0	58.6	9.7	7.2
	Sum	136.3	150.4	138.8	88.6	62.1	36.0
Lys102	Glu24	0.0	0.0	0.0	0.0	4.4	4.5
	Glu59	0.0	0.0	0.0	0.0	0.0	0.0
	Asp60	0.5	0.3	0.4	0.2	37.3	29.6
	Glu63	0.0	0.0	0.0	0.0	1.5	0.6
	Glu64	0.0	0.1	0.0	0.1	59.1	54.0
	Glu98	51.2	47.2	44.9	58.7	19.4	6.9
	Glu99	0.0	0.0	0.0	0.0	6.8	4.6
	Sum	51.7	47.5	45.3	58.9	128.5	100.2
Average^a^		82.5	83.6	79.7	77.7	79.0	74.0
(B)							
Lys19	Glu46	0.0	0.1	0.0	0.0	1.1	0.1
	Glu47	72.3	69.1	91.6	57.4	35.2	32.2
	Sum	72.3	69.2	91.6	57.4	36.3	32.3
Lys23	Glu42	0.0	0.0	0.0	0.0	0.2	0.0
Arg25	Glu42	0.0	0.0	0.0	0.0	2.3	0.8
Arg33	Glu34	0.0	0.7	3.7	0.0	66.5	38.3
	Glu47	0.0	0.0	0.0	0.0	0.0	0.0
	Sum	0.0	0.7	3.7	0.0	66.5	38.3
Arg36	Glu15	33.2	53.1	64.9	34.3	34.1	13.1
	Glu34	0.1	0.0	0.1	0.4	3.4	3.2
	Sum	33.3	53.1	64.9	34.7	37.5	16.3
Lys44	Glu15	0.0	0.0	0.0	0.0	0.1	0.0
Lys56	Glu50	0.2	12.6	0.3	0.0	59.7	13.9
Lys66	Asp87	30.3	46.0	51.8	63.5	25.8	11.1
	Glu90	44.9	9.6	12.4	17.1	28.6	36.5
	Asp91	0.0	0.0	0.0	0.0	0.0	0.1
	Sum	75.2	55.5	64.2	80.6	54.4	47.7
Lys70	Glu90	2.2	1.2	1.1	18.6	10.1	9.5
	Asp91	57.1	11.0	22.6	44.1	58.5	56.4
	Sum	59.3	12.1	23.7	62.7	68.6	65.9
Arg82	Asp86	24.8	98.0	99.8	57.9	90.3	8.5
	Asp87	19.3	71.7	70.1	45.9	39.5	88.2
	Sum	44.1	169.8	169.9	103.8	129.8	96.8
Lys101	Glu42	0.0	0.0	0.0	0.0	0.3	0.3
Lys102	Glu42	0.0	0.0	0.0	0.0	0.4	0.1
Average^a^		23.7	31.1	34.9	28.3	38.0	26.0

^a ^Average values per positively charged residue.

As described in the previous section, Arg68 formed a strong salt bridge with Glu24 and Glu71 in simulation using Charmm27_tip3p, Amber99sb_spc/e, and Amber99sb_tip3p (Fig. S3A). On the other hand, when using Amber14sb_tip3p, Gromos43a1_spc/e, and Gromos53a6_spc/e, Arg68 formed salt bridges with Glu64 and Glu67 as well as Glu24 and Glu71, although their percent occupancies were reduced (Table 2A). These results can be observed in snapshots of PhCutA1 acquired in 100‐ns MD simulations at 300 K. As shown in Fig. S4, in Amber99sb_tip3p, Arg68 interacted strongly with Glu24 and Glu71, and these interactions remained stable over 400 ns. In the case of Amber14sb_tip3p, Arg68 interacted strongly with Glu24 and Glu64 at 100 ns, but the rotational isomer of Arg68 located in the middle of α‐2 helix could easily interact with Glu71 (Table S7). These tendencies were also observed in simulations using Gromos43a1_spc/e and Gromos53a6_spc/e (Table 2A).

Arg82 of PhCutA1 formed strong salt bridges with many favorable ion pairs due to intra‐ and inter‐subunit interactions. In the crystal structure of the trimer (A, B, and C subunits of 4nyo), Arg82 was close to Glu59, Glu63, and Asp84 in the same subunit and to Asp86 and Asp87 in another subunit (Fig. S5A). Many negatively charged residues are located near Arg82, and these ion–ion interactions fluctuated strongly during 400‐ns MD simulations. In simulations using Charmm27_tip3p, Amber14sb_tip3p, and Gromos53a6_spc/e, Arg82 remained engaged in a strong interaction with Asp84, but when using the other force fields, the occupancies were quite small (Table 2A). Because it is difficult to alter the movement of Arg82, the difference in this interaction might be due to fluctuation of Asp84. Occupancies of salt bridges between Arg82 and Glu59 decreased to 28–59%, in contrast to the strong interactions in the crystal structures, indicating that this effect might be caused by fluctuation of Glu59, which is located in a loop region. On the other hand, the inter‐subunit interactions of Arg82 with Asp87 in the crystal structure exchanged partners (Asp86 or Asp87) depending on the force fields used in the MD simulations. The total occupancy of the inter‐subunit interaction was lowest when using Charmm27_tip3p (44%) (Table 2B), but highest when using Amber99sb_spc/e or Amber99sb_tip3p (170%). On the other hand, Gromos43a1_spc/e and Gromos53a6_spc/e differed in terms of the favorable pairs (Asp86 and Asp87) engaged in interactions with Arg82 (Table 2B). This difference among force fields was also apparent in the intra‐subunit interaction between Arg82 and Asp84: the occupancies were 7% and 87% in the case of Gromos43a1_spc/e and Gromos53a6_spc/e, respectively. Figure S5B shows a snapshot of the configuration around Arg82 at 200 ns in a simulation using Gromos53a6_spc/e. The difference within the Gromos group might be correlated with the difference in *R*_g_ (Fig. 2B and Table 1). Overall, these results indicate that ion–ion interactions in the crystal structure, in which negatively charged residues crowd around Arg82, are stronger than those in solution.

The amino group of the N‐terminal residue Met1 in the crystal structure formed a salt bridge with Glu59, but Asp84 and Asp86 in the loop region also formed salt bridges with the N terminus during 400‐ns MD simulations. As shown in Fig. S6, in the case of Amber99sb_tip3p, the N terminus formed tight salt bridges with Asp84. The occupancies of the interaction between the N terminus and Glu59 were lower for Amber99sb_spc/e and Amber99sb_tip3p than for the other four force fields, whereas those of Asp84 were higher (Table 2A). The occupancy of Asp84 was only 2.7% in simulations using Amber14sb_tip3p. In the case of Gromos43a1_spc/e and Gromos53a6_spc/e, the N terminus also forms salt bridges with Asp84 and Asp86, despite the almost 100% occupancy of Glu59. Because these residues interacting with the N terminus are not involved in α‐helix or β‐sheet, dependence on force fields might be strengthened.

Lys44 formed salt bridges with Glu42 and Glu46, which are located in a loop region between the β‐2 and β‐3 sheets. As shown in the snapshots in Fig. S7A,B, in the case of Charmm27_tip3p, Lys44 formed strong salt bridges with both residues, whereas in the case of Gromos53a6_spc/e, Glu42 was far from Lys44, and the occupancy of salt bridges was only 1.8%. The sum of occupancy of Ly44 was lowest for Amber14sp_tip3p (41%) and highest for Charmm27_tip3p (85%) (Table 2A). On the other hand, in simulations using Gromos43a1_spc/e and Gromos53a6_spc/e, Lys44 formed salt bridges with Glu47 with occupancies of 1.8% and 0.5%, respectively, although these values are not large. The expansion of flexibility in the loop region in the cases of Gromos43a1_spc/e and Gromos53a6_spc/e was also observed in the case of the interaction with Asp84 or Asp86, described above in the discussion of the N terminus.

Lys70 formed salt bridges with Glu67 in the same subunit and with Glu90 and Glu91, which are located in the N terminus of the α‐3 helix in another subunit. In regard to intra‐subunit interactions, the occupancies of salt bridges between Lys70 and Asp76 were 18% and 16%, respectively, in the cases of Amber99sb_spc/e and Amber99sb_tip3p, but < 1% in simulations using the other force fields. In terms of inter‐subunit interactions, in the cases of Amber99sb_spc/e and Amber99sb_tip3p, the sum of occupancy of Lys70 was 12% and 24%, respectively, whereas for other force fields the value ranged from 60% to 69%. The distances between Lys70 and Glu90 or Glu91 were quite close in the case of Gromos43a1_spc/e (Fig. S8A), but in Amber99sb_tip3p, they were far (Fig. S8B). That is, the N terminus of the α‐3 helix was far from Lys70 in the adjacent subunit in the case of Amber99sb_tip3p, suggesting a difference in inter‐subunit interactions that can be attributed to the use of different force fields. Consequently, Lys70 had the opportunity to form a salt bridge with Asp76 in the same subunit when using Amber99sb_spc/e and Amber99sb_tip3p. This observation is correlated with the difference in *R*_g_, which was larger for Amber99sb_spc/e and Amber99sb_tip3p than for the other force fields (Fig. 2B).

In the crystal structures, Arg36 in the middle of the β‐2 strand formed a tight salt bridge with Glu15, located in the middle of the α‐1 helix in another subunit. Arg36 also formed a salt bridge with Glu47 in the middle of the β‐3 strand in the same subunit. Figure S9 shows snapshots of Arg36 in the cases of Amber99sb_tip3p and Gromos53a6_spc/e at 200 ns. The occupancies of the Arg36–Glu15 interaction were higher with Amber99sb_spc/e and Amber99sb_tip3p than the other force field; in particular, the occupancy of inter‐subunit salt bridges in between Arg36 and Glu15 was lowest for Gromos53a6_spc/e among the six force fields. On the other hand, the sum of occupancy of Arg36 in the same subunit was the highest (99%) for Charmm27_tip3p and the lowest (59%) for Amber14sb_tip3p. In the case of Arg36, it is quite difficult to explain the difference in the strength of salt bridges among the six force fields, although one possibility is difference in the combination of rotamers (Table S7).

Lys101 and Lys102 are located in the C terminus of PhCutA1. In the cases of Charmm27_tip3p, Amber99sb_spc/e, and Amber99sb_tip3p, Lys101 formed salt bridges with Glu64 with percent occupancies above 80%, whereas in the case of Gromos43a1_spc/e and Gromos53a6_spc/e, the corresponding values were only 7–8% (Table 2A). On the other hand, in the cases of Gromos43a1_spc/e and Gromos53a6_spc/e, Lys102 formed a salt bridge with Glu64 (occupancies of 54–60%), but the occupancies were barely detectable when using the other force fields. Figure S10A,B shows typical snapshots at 100 ns in simulations using Charmm27_spc/e and Gromos53a6_spc/e, respectively. The two amino groups of Lys102 were located in opposite directions in simulations using these two force fields, as shown in the figure. In the cases of Gromos43a1_spc/e and Gromos53a6_spc/e, Lys102 forms salt bridges with several favorable residues in addition to Glu64 and Glu98, whereas in the cases of the other force fields, Lys102 only forms a salt bridge with Glu98 (Table 2A). These results indicate that the two C‐terminal residues fluctuate much more intensely in the two Gromos force fields than in the other cases (Fig. S2B).

Arg58, located in a small loop between the β‐3 sheet and 3_10_‐helix, formed salt bridges with Glu60 with occupancies of 80–100% and with the C‐terminal carboxyl group (Lys102) with occupancies of 17–47%. For both salt bridges, the occupancy was lowest for Gromos43a1_spc/e, which might be related to the flexibility of loop region. The snapshots of the C terminus and Arg58 at 200 ns in simulations using Amber14sb_tip3p and Gromos43a1_spc/e are shown in Fig. S11A,B, respectively.

Lys56 formed a salt bridge with Glu50 in another subunit in simulations using Gromos43a1_spc/e (Fig. S12A), but not when using Charmm27_tip3p, Amber99sb_tip3p, or Amber14sb_tip3p (Fig. S3B and Table 2B). A snapshot of the inter‐subunit interaction between Lys56 and Glu50 (Fig. S12B) in a simulation using Amber99sb_spc/e shows that Glu50 was stably located in the middle of the β‐2 sheet, although the salt bridge was longer than in a simulation using Gromos43a1_spc/e (0.69 vs. 0.33 nm). On the other hand, in the case of Gromos43a1_spc/e (Fig. S12A), the β‐2 sheet around Glu50 was absent. The fluctuation (instability) in the middle of β‐2 sheet might create the opportunity to form a salt bridge < 0.6 nm in length.

### Re‐evaluation of the electrostatic energies of charged residues for PhCutA1

The electrostatic energies of charged residues for PhCutA1 have been estimated based on the crystal structures in order to evaluate the contribution of charged residues to conformational stability. In terms of unfavorable interactions, the worst three residues are Asp86, Glu12, and Arg33, with ion–ion interaction energies of 19.6, 11.6, and 10.9 kJ·mol^−1^, respectively 17. As shown in Fig. S13A, in the crystal structure Asp86 interacts repulsively with Asp84 in another subunit, and the distance between Asp86 and Asp84 ranges from 0.34 to 0.41 nm. However, this repulsion energy was weakened during MD simulations at 300 K: the average electrostatic energy for Asp86 became 6.5 kJ·mol^−1^ in the case of Gromos43a1_spc/e, as compared to 19.6 kJ·mol^−1^ in the crystal structure (Table S4). A snapshot of the configuration around Asp86 confirmed that the distances between Asp86 and Asp84 in the adjacent subunit increased from 0.45 to 0.74 nm (Fig. S13B). In the cases of Charmm27_tip3p and Amber99sb_spc/e, the electrostatic energies for Asp86 were 16.5 and 4.5 kJ·mol^−1^, respectively (Table S4).

Glu12 engages in repulsive interactions with Asp10 and Glu15. During 400‐ns MD simulations, the electrostatic energies of Glu12 became 8.2, 7.9, and 6.5 kJ·mol^−1^ for Gromos43a1_spc/e, Charmm27_tip3p, and Amber99sb_spc/e, respectively, indicating slight relaxations.

In X‐ray crystal structure of PhCutA1 (PDB ID 4nyo), Arg33 is completely buried in the interior of the molecule. However, the side chain of Arg33 appeared on the surface of the tetramer in MD simulations using Gromos43a1_spc/e (Fig. S14B), whereas in the case of Amber99sb_tip3p, this residue was still located in the interior of the trimer. The superimposed structure shown in Fig. S14B suggests that both structures are rotational isomers of Arg33 (Table S7). On the surface, the ionic group of Arg33 can form ion–ion interactions with Glu34 residues in the same or adjacent subunits (Fig. S14A). Except for Gromos43a1_spc/e and Gromos53a6_spc/e, occupancies of salt bridges were not detected in intra‐ or inter‐subunit interactions (Tables 2A,B), indicating that the side chains of Arg33 remain buried in the interior of the trimer during 400‐ns MD simulations. When the ionic groups of charged residues are buried in the interior of protein molecules, the difference in strengths of salt bridges among force fields might stand out between Gromos groups with the united‐atom representation and other groups with the all‐atom representation. During 400‐ns MD simulations, the electrostatic energy of Arg33 significantly improved to −4.9 kJ·mol^−1^ in the case of Gromos43a1_spc/e and to 5.8 and 2.3 kJ·mol^−1^ in the cases of Charmm27_tip3p and Amber99sb_spc/e, respectively (Table S4). The adjustment of unfavorable energy for Arg33 was the highest for Gromos43a1_spc/e.

**Figure 4:**
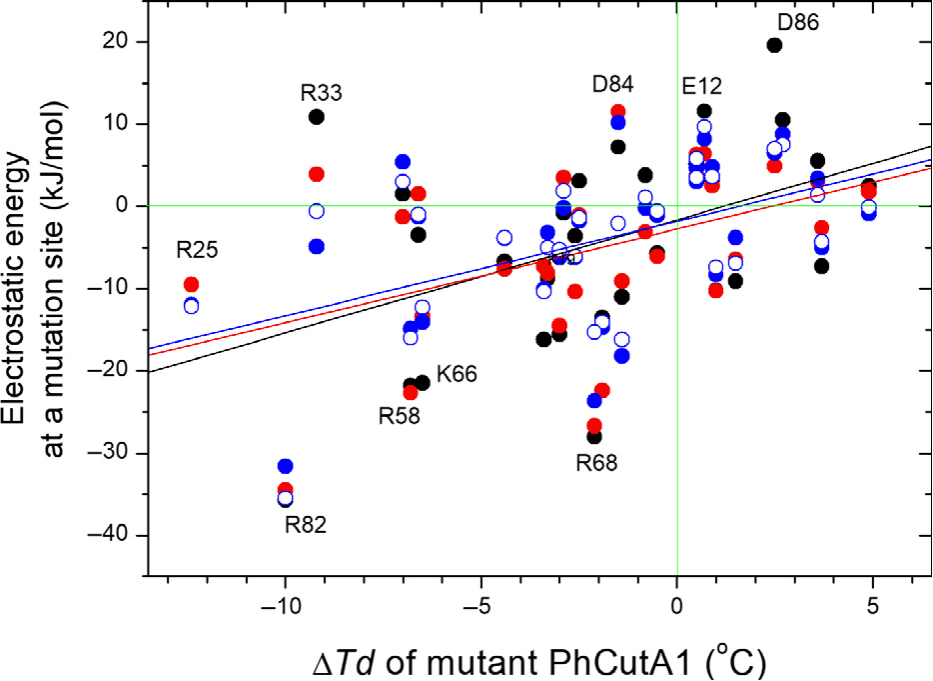
Correlation between electrostatic energy at mutation sites estimated from FoldX using different structures and difference in denaturation temperatures due to mutation of PhCutA1. Black represents the structure obtained from ABC subunits from 4nyo (crystal structures). Red, blue, and open circles represent from structures in MD simulations using Amber99sb_tip3p, Gromos43a1_spc/e, and Gromos53a6_spc/e, respectively. All data are listed in Table S4. Linear lines of black, red, and blue represent linear regressions of the data for ABC subunits from 4nyo, Amber99sb_tip3p, and Gromos53a6_spc/e, respectively.

Figure 4 shows the correlation between electrostatic energy at mutation sites estimated by FoldX using various structures and the difference in denaturation temperatures (Δ*T*_d_) 17 due to mutations of charged residues to noncharged residues. As mentioned above, the figure shows that the electrostatic energies at Asp86 and Arg33 are significantly reduced in structures obtained from MD simulations. Detailed data are provided in Table S4. On the other hand, in the cases of Gromos43a1_spc/e and Gromos53a6_spc/e, the electrostatic energies at Arg58, Lys66, and Arg68 are higher than those in the crystal structures (Fig. 4 and Table S4). These data indicate that the electrostatic energies of structures from MD simulations approach the line generated by regressing electrostatic energies at mutation sites vs. Δ*T*_d_ due to mutations (Fig. 4). Through molecular adaptation, changes in conformational stability due to mutations should optimize factors such as hydrophobic interactions, electrostatic interactions, and entropic effects. Consequently, Δ*T*_d_ and electrostatic energies at mutation sites should not exhibit a strict linear correlation. However, our data show a weak linear correlation between them (Fig. 4 and Table 3); because the energies of ion–ion interaction with MD simulation of Gromos group approach nearer the linear regression lines than those of the crystal structures, we can conclude that the configurations of charged residues in structures obtained by MD simulations using the Gromos group are better than those obtained with other force fields. Table 3 shows a comparison of linear regression error coefficients between electrostatic energy at a targeted residue and the difference in denaturation temperature among eight different structures. The error coefficients of Gromos group were lower than those of the others, suggesting that the configurations of charged residues in MD simulations performed using the Gromos group are superior.

**Table 3:** Linear regression error coefficients between electrostatic energy at targeted residue and difference in denaturation temperature of mutant PhCutA1 among different structures. R, SD, and *P* represent correlation coefficient, standard deviation, and *P* value, respectively, in linear regression using data of Table S4. Three lines in Fig. 4 are linear regressions between Δ*T*_d_ and electrostatic energy for three structures described.

	*R*	SD	*P*
Crystal structures			
ABC of 4nyo	0.465	11.2	0.008
DEF of 4nyo	0.483	10.5	0.006
Force fields in MD simulation			
Charmm27_tip3p	0.464	10.0	0.009
Amber99sb_spc/e	0.442	9.6	0.013
Amber99sb_tip3p	0.445	9.8	0.012
Amber14sb_tip3p	0.477	10.1	0.007
Gromos43a1_spc/e	0.498	8.6	0.004
Gromos53a6_spc/e	0.522	8.0	0.003

### The interaction of charged residues with counter‐ions of salts

In the present MD system, salt concentration (NaCl) was set to 150 mm. The percent occupancies of salt bridges (< 0.6 nm) of positively charged residues in PhCutA1 with chloride (Cl^−^) ion were examined (Table 4). In the case of Charmm27_tip3p and Amber14sb_tip3p, Arg33 which was completely buried in the interior of a molecule in the initial state formed a salt bridge with one Cl^−^ ion by almost 100%, and salt bridges of its Arg33 with negatively charged residues were not observed (Table 4). As mentioned above, in the case of Gromos43a1_spc/e, Arg33 which was located on the surface formed a salt bridge with a negatively charged residue by percent occupancy of 121.3%, but percent occupancy (summation) of salt bridges of its Arg33 with several Cl^−^ ions was 13.2% (Table 4). Trajectories of distance between Arg33 in PhCutA1 and Cl^−^ ion during 400‐ns MD simulations at 300 K for three force fields are shown in Fig. S15. In the case of Charmm27_tip3p and Amber99sb_tip3, percent occupancies of salt bridges of Arg33 with one Cl^−^ ion were 100.0% and 86.0% during 400‐ns MD simulations, respectively. In the case of Gromos43a1_spc/e, that was only 1.7% (Fig. S15): the summation of occupancies of Arg33 with several Cl^−^ ions was 13.2% as mentioned above. These results suggest that charged residues which are completely buried in a molecule are neutralized by counter‐ions.

**Table 4:** Percent occupancies of salt bridges (< 0.6 nm) of positively charged residues in PhCutA1 with Cl^−^ ions during 400‐ns MD simulations at 300 K using the indicated force fields. Highlight shows the interaction with Arg33.

Positively charged Residues	Force fields											
	Charmm27 _tip3p		Amber99sb _spc/e		Amber99sb _tip3p		Amber14sb _tip3p		Gromos43a1 _spc/e		Gromos53a6 _spc/e	
	N. C. Residue^a^	Cl^−^ ion	N. C. Residue^a^	Cl^−^ ion	N. C. Residue^a^	Cl^−^ ion	N. C. Residue^a^	Cl^−^ ion	N. C. Residue^a^	Cl^−^ ion	N. C. Residue^a^	Cl^−^ ion
N‐terminal	111.8	0.4	129.2	1.7	102.7	2.1	97.7	0.8	137.3	0.6	127.3	0.2
Lys16	30.6	10.1	30.7	7.8	35.0	5.9	41.6	6.1	55.1	4.0	48.6	4.7
Lys19	162.7	7.4	163.3	4.2	187.0	2.6	149.2	4.2	126.4	4.1	122.4	5.2
Lys23	4.3	14.7	1.2	13.1	1.4	8.4	7.5	9.8	6.6	8.3	8.4	10.2
Arg25	97.3	2.3	96.2	1.7	91.3	1.3	99.4	1.5	92.4	0.9	96.8	0.4
Arg33	0.0	99.9	1.6	58.8	3.9	62.1	0.0	99.8	121.3	13.2	73.5	18.0
Arg36	132.0	5.8	133.9	2.0	147.3	1.4	93.8	3.6	104.8	6.3	87.6	8.5
Lys44	84.5	6.7	62.0	6.9	54.7	5.6	40.6	5.5	48.6	6.4	62.7	6.2
Lys49	90.3	10.4	122.5	4.6	96.1	3.8	60.2	7.0	134.9	7.2	83.2	10.4
Arg58	132.5	1.1	134.4	0.4	130.6	0.3	147.0	0.5	102.4	2.6	111.5	1.8
Lys66	135.0	2.0	139.7	1.6	146.0	1.4	177.8	0.9	144.9	0.6	102.9	0.9
Arg68	193.9	4.9	195.5	1.9	195.6	2.2	208.5	2.3	143.7	1.0	117.0	1.6
Lys70	92.1	3.0	85.7	1.7	87.8	1.9	106.3	1.9	123.8	0.9	95.0	1.5
Arg82	171.6	1.6	218.1	0.6	220.9	1.0	230.3	1.2	188.4	0.4	242.6	0.4
Lys94	60.7	6.2	82.7	3.0	89.3	3.1	53.4	4.3	78.0	3.6	53.6	3.6
Lys101	136.3	3.0	150.4	1.7	138.8	1.7	88.6	3.0	62.4	3.0	36.4	3.5
Lys102	51.7	6.3	47.5	4.7	45.3	4.5	58.9	4.3	128.9	2.0	100.4	2.0
average1^b^	99.3	10.9	105.6	6.8	104.4	6.4	97.7	9.2	105.9	3.8	92.4	4.6
average2^b^	105.5	5.4	112.1	3.6	110.6	3.0	103.8	3.6	104.9	3.2	93.5	3.8

^a ^N. C. Residue represents percent occupancies with Negatively Charged Residues. Data come from Table 2A,B. ^b ^average1 and average2 represent the average of 17 positively charged residues and that of 16 residues except for data of Arg33, respectively.

Except for Arg33, the average percent occupancy of intra‐ and inter‐subunit salt bridges for each positively charged residues ranged from 93.5% to 112.1% among the six force fields (average 2 in Table 4), and the average value was 105.1 ± 6.6%. On the other hand, the value of interactions with Cl^−^ ions ranged from 3.0% to 5.4% (average 2 in Table 4) with mean of 3.8 ± 0.8%. The average values of the interaction between charged residues of amino acids were greater by about 30 times than others, suggesting that the ion interaction between amino acids is considerably stronger than that of charged residues with counter‐ions of salts. The big difference in the interaction of charged residues with counter‐ions on the surface of a molecule was not detected among six force fields.

Furthermore, the average percent occupancies of intra‐ and inter‐subunit salt bridges for each negatively charged residues with Na^+^ ions were also examined: They were from 60.2% to 69.7% among the six force fields (Table S8), and the average value was 65.0 ± 4.0%. The big difference between the average percent occupancies between negatively and positively charged residues (Table 4 and Table S8) is caused by the difference in residue numbers between them. On the other hand, the value of interactions of negatively charged residues with Na^+^ ions ranged from 19.4% to 59.6% (Table S8) with mean of 31.9 ± 16.0%. This bigger value compared with that of Cl ions suggests the neutralization of excess negatively charged residues.

### Re‐evaluation of salt bridges obtained from X‐ray crystal structures of PhCutA1

Figure S3 also shows the distance of salt bridges < 0.6 nm, obtained from X‐ray crystal structures of PhCutA1. The 4nyo.pdb structure of PhCutA1 was obtained in normal buffer solutions, whereas the 1umj.pdb structure of the same protein was obtained in the presence of 3 m guanidine hydrochloride, a denaturant 39. PhCutA1 is not denatured in 3 m guanidine hydrochloride 39, 40, but the local structures on the surface of the protein, including salt bridges, might be perturbed by such severe conditions. Focusing on the main pair residues (strong salt bridges) with positively charged residues in Fig. S3, the pair residues of salt bridges observed from six kind MD simulations coincided with those from three kind crystal structures except for three pairs, which are Lys49 and Lys94 in ABC subunits of 4nyo, and Lys102 in three crystals. Ion pairs of Lys49 with Glu34 and Lys94 with Glu91 in crystal structures coincided with those of MD simulations except for ABC subunits of 4nyo, in which B factors (suggesting fluctuation of crystal atoms) were also high (Fig. S3A), indicating that the pairs involving Lys49 and Lys94 in ABC of 4nyo are questionable. Lys102, a C‐terminal residue with a high B factor, is not detectable in 1umj, which was crystallized in the presence of 3 m guanidine hydrochloride 40. These results indicate that if the crystal data are probed strictly, the main pairs of salt bridges obtained from MD simulations at 300 K completely agree with X‐ray crystal data measured at 100 K. However, charged residues in solution constantly fluctuate, as shown in Fig. 3, and would have many chances to form salt bridges with other favorable ionic pairs.

### Difference in the strengths of salt bridges of PhCutA1 using six force fields

Next, we counted the numbers of ion pairs forming salt bridges < 0.6 nm in length during 400‐ns MD simulations for 186 targeted intra‐subunit interactions and 60 targeted inter‐subunit interactions. Table 5 lists the numbers and percentages of salt bridges vs. percent occupancy. The number of ion–ion interactions with percent occupancies > 0.1% was 106 of 186 for Charmm27_tip3p and 161 of 186 for Gromos43a1_spc/e (57% and 87%, respectively). These results indicate that in the case of Gromos43a1_spc/e, PhCutA1 forms much more favorable salt bridges than in the other cases. On the other hand, occupancies > 90% were observed for 21 salt bridges in the case of Charmm27_tip3p and only six in the case of Gromos43a1_spc/e (Table 5A), indicating that the salt bridges of Charmm27_tip3p at each site are more stable than those of other force fields, although the average values of occupancy for each positively charged residue were similar (Table 2).

**Table 5:** (A) Number of targeted ion–ion interactions forming intra‐subunit salt bridges in simulations using the indicated force fields. (B) The number of targeted ion–ion interactions forming inter‐subunit salt bridges in simulations using the indicated force fields. Data represent number and percentage of targeted ion–ion interactions forming salt bridges (< 0.6 nm).

%^a^	Force fields											
	Charmm27_tip3p		Amber99 _spc/e		Amber99_tip3p		Amber14_tip3p		Gromos43a1_spc/e		Gromos53a6 _spc/e	
	Number	%^b^	Number	%^b^	Number	%^b^	Number	%^b^	Number	%^b^	Number	%^b^
(A)												
> 0.1	106	57.0	115	61.8	114	61.3	118	63.4	161	86.6	142	76.3
> 1	94	50.5	96	51.6	98	52.7	94	50.5	135	72.6	121	65.1
> 10	67	36.0	74	39.8	70	37.6	71	38.2	81	43.5	77	41.4
> 40	40	21.5	51	27.4	46	24.7	48	25.8	45	24.2	40	21.5
> 70	29	15.6	26	14.0	25	13.4	20	10.8	15	8.1	18	9.7
> 80	28	15.1	24	12.9	21	11.3	17	9.1	10	5.4	15	8.1
> 90	21	11.3	17	9.1	14	7.5	15	8.1	6	3.2	10	5.4

%^a^	Force fields											
	Charmm27_tip3p		Amber99 _spc/e		Amber99_tip3p		Amber14_tip3p		Gromos43a1_spc/e		Gromos53a6 _spc/e	
	Number	%^c^	Number	%^c^	Number	%^c^	Number	%^c^	Number	%^c^	Number	%^c^
(B)												
> 0.1	28	46.7	30	50.0	28	46.7	26	43.3	48	80.0	46	76.7
> 1	24	40.0	27	45.0	22	36.7	24	40.0	39	65.0	34	56.7
> 10	21	35.0	21	35.0	18	30.0	14	23.3	28	46.7	26	43.3
> 40	8	13.3	15	25.0	16	26.7	2	3.3	13	21.7	8	13.3
> 70	2	3.3	7	11.7	10	16.7	0	0	6	10.0	5	5.0
> 80	1	1.7	3	5.0	8	13.3	0	0	5	8.3	5	5.0
> 90	0	0	3	5.0	5	8.3	0	0	3	5.0	0	0

^a ^Occupancy of each ion–ion interaction. ^b ^Percentage of 186 targeted ion–ion interactions. ^c ^Percentage of 60 targeted ion–ion interactions.

In regard to inter‐subunit interactions (Table 5B), Amber14_tip3p had the lowest percent occupancy (> 0.1) and Gromos43a1_spc/e the highest. In the case of higher occupancies, the data for the inter‐subunit interactions were more complicated due to differences in the strengths of subunit–subunit interactions among different force fields. However, in the case of the Gromos group, charged residues of PhCutA1 might be able to interact with many more favorable charged residues, both within and between subunits, than in the case of the other force fields.

## Concluding remarks

1. We investigated the influence of six different force fields on the formation of salt bridges involving positively charged residues (Arg or Lys) and favorable partners in MD simulations of the PhCutA1 trimer. The force fields used were Charmm27_tip3p, Amber99sb_spc/e, Amber99sb_tip3p, Amber14sb_tip3p, Gromos43a1_spc/e, and Gromos53a6_spc/e.
2. We examined the effects of force fields on RMSD, RMSF, *R*_g_, and secondary structures. The average RMSD was lowest for Charmm27_tip3p and similar for the other force fields. *R*_g_ was lowest for Gromos43a1_spc/e and highest for Amber99sb_spc/e and Amber99sb_tip3p. The average number of residues in each type of secondary structures was similar among the six force fields, but percent helicity was highest for Charmm27_tip3p.
3. Percent occupancies of salt bridges < 0.6 nm for all the targeted pairs of 246 residues (186 residues for intra‐ and 60 ones for inter‐subunit interactions) (Fig. S3) and for all positively charged residues (Table 2) were used to estimate the strengths of salt bridges. Furthermore, the average length with its error bar of salt bridges suggests that its average value considerably fluctuates during 400‐ns MD simulations (Table S3).
4. The average percent occupancy of intra‐subunit salt bridges for each positively charged residue ranged from 83.6% for Amber99sb_spc/e to 74.0% for Gromos53a6_spc/e; the average was 79.4 ± 2.5% (Table 2A). For inter‐subunit interactions, the value ranged from 38.0% for Gromos43a1_spc/e to 23.7% for Charmm27_spc/e; the average was 30.3 ± 4.3% (Table 2B). Thus, the average strength of salt bridges for positively charged residues did not differ significantly among the six force fields. However, in the case of the Gromos group, positively charged residues of PhCutA1 were able to interact with many more favorable residues (Glu or Asp) in the other force fields, and this was true for both intra‐ and inter‐subunit interactions (Table 5). On the other hand, the strengths of salt bridges at specific sites within structures were significantly affected by the force field used.
5. The orientation of the ionizable group of a charged residue is determined by the rotational isomer. For example, in the α‐2 helix, a rotational isomer of Arg68 forms a salt bridge with Glu64 in the cases of Charmm27_tip3p, Amber99sb_spc/e, and Amber99sb_tip3p or with Glu71 in the other cases (Table S7). The side chain of Arg33 in the β‐2 sheet is buried in the interior of the molecule in the initial structure, but during MD simulations using Gromos43a1_spc/e and Gromos53a6_spc/e, the side chain (which is located on the protein surface) rotates to form salt bridges with Glu34 in the same and neighboring subunits. By contrast, in the other cases, Arg33 remains buried in the interior of the molecule. Arg82 forms a salt bridge with Asp84 in the cases of Charmm27_tip3p, Amber14sb_tip3p, and Gromos53a6_spc/e, but not otherwise. For Arg82, this difference seems to be caused by fluctuation (isomerization) for Glu84 of the pair residue. It is possible that the charged residues fluctuate over all possible rotational isomers in solution, depending on the differences in the energy levels of rotamer among the six force fields.
6. The occupancies of a salt bridge between Lys70 and Glu90 in the inter‐subunit interaction were smaller in the cases of Amber99sb_spc/e and Amber99sb_tip3p than the other force fields. This might be correlated with the larger *R*_g_ values (i.e., loose structures) obtained with Amber99sb_spc/e and Amber99sb_tip3p.
7. Lys102 forms salt bridges with five different residues in the cases of Gromos43a1_spc/e and Gromos53a6_spc/e, but with only two residues in the other force fields. The same effects were observed for many other interactions, such as N terminus–Asp86 and Lys101–Glu98, which are located in loop regions. The greater flexibility of side chains of charged residues in the case of the Gromos group might be correlated with differences in the atomic representations of aliphatic CH_n_ (i.e., united vs. all‐atom representation).
8. The occupancies of salt bridges for Amber99sb_spc/e and Amber99sb_tip3p were similar, indicating that the choice of water model barely affects the strength of salt bridges in the case of the Amber99sb force field.
9. The electrostatic energies of charged residues at mutation sites were re‐evaluated for two structures obtained by X‐ray crystal analysis and six structures obtained by MD simulations. From the correlation between electrostatic energies at mutation sites and the difference in denaturation temperatures of mutants in which charged residues were replaced with noncharged residues, the configurations of charged residues in structures in simulations using Gromos43a1_spc/e and Gromos53a6_spc/e were superior to those in the six other structures.
10. If the crystal data were probed strictly, the main pairs of salt bridges obtained from MD simulations at 300 K completely agreed with the X‐ray crystal data measured at 100 K, although their strengths differed among the six force fields. However, many unfavorable interactions with high repulsive energies were observed in the crystal structures. These unfavorable interactions were diminished during MD simulations.
11. Finally, in the case of the Gromos group, positively charged residues could interact with many more favorable residues (Glu or Asp) than in the other force fields, especially in loop regions, causing the apparent strength at each site to be weaker. The strength at each site was highest for Charmm27_tip3p. That is, the Gromos group has the advantage that a charged residue has more opportunities to seek for favorable interaction partners in solution during the limited period of the MD simulation, although this notion requires confirmation.

## Materials and methods

For MD simulations, we used the trimer structure of PhCutA1, which contains three identical subunits (A, B, and C subunits; PDB ID 4nyo) (Fig. 1). Buried ratio and pKa of negatively and positively charged residues in the crystal structure (4nyo) of PhCutA are listed in Table S9.

Molecular dynamic simulations were performed using the gromacs software (ver. 4.5.5) 41, 42 at 300 K, using running conditions as described 15, 19. In this study, we compared the structures of PhCutA1 in MD simulations using six force fields (listed in Table 1). Hydrogen atoms were added to each protein. The models were solvated in water boxes with a minimum distance of 1.2 nm between the protein and the box. Counter‐ions were added to the model to neutralize any net charge. Salt concentration was set to 150 mm. The number of Na^+^, Cl^−^, and H_2_O in the simulation box and size of the box during MD simulations are listed in Table S10. The periodic boundary condition was adopted and the long‐range electrostatic interactions were computed using the particle mesh Ewald method 43. The system was weakly coupled to a heat bath by velocity rescaling 44 with a relaxation time of 0.1 picosecond (ps). A Parrinello–Rahman barostat 45 was used to maintain a pressure constant at 1 atm with a relaxation time of 0.5 ps. Hydrogen atoms were constrained using LINCS 46, and MD simulations at 300 K were conducted with an integration time step of 1 fs. Energy minimizations were done to remove bad van der Waals contacts. Next, the temperature was raised from 50 to 300 K in increments of 50 K, with 10 000 integration steps at each temperature and a harmonic constraint of C‐alpha atoms. Thereafter, the ensemble was equilibrated through four 100‐ps cycles with gradually released harmonic constraints: 1000, 100, 10, and 1 kJ mol^−1 ^nm^−2^. The subsequent MD stages for the PhCutA1 were carried out without any restraint at 300 K. The resultant MD trajectories were analyzed using the gromacs software, as described previously 15, 19. For estimation of salt bridges, distance was calculated (using the command ‘gmx distance’) between the C_ε_ atom of Lys (or C_ζ_ of Arg) and the C_γ_ atom of Asp (or C_δ_ of Glu).

We confirmed how robust the results for the formation of salt bridges are (a) within the single force field during 400‐ns MD simulations and (b) when different initial geometry is introduced. In the case of (a), percent occupancies of intra‐subunit salt bridges in PhCutA1 were examined at each 100‐ns during 400‐ns MD. As shown in Table S5, the robustness in the single force field was confirmed except for the early stage (< 100‐ns) of Amber99sb_spc/e. In the case of (b), percent occupancies of intra‐subunit salt bridges for each of three identical subunits in PhCutA1 were examined during 400‐ns MD at 300 K using the six force fields. As shown in Table S6, the standard deviation of average values among three subunits was < 2.1% in the six force fields, suggesting the robustness of our results among the different initial structures.

We also examined the validation for the different population of the rotamers in employed force fields for residues involved in salt bridges presented on Fig. S4–S14 using a software MolProbity (http://molprobity.biochem.duke.edu). As shown in Table S7, the most residues examined show rotamers of the favored region, indicating validated conformation preferences of residues involved in the bonds for the studied X‐ray and MDs models.

To evaluate the energy of ion–ion interactions in PhCutA1, we used the algorithm FoldX 47, which can quantitatively estimate the factors that are important for protein stability. FoldX is available via a web interface at http://foldxsuite.crg.eu. Electrostatic energies due to ion–ion interactions between charged residues were calculated using the ‘AllAtoms_Electro’ file in FoldX. The electrostatic energy in FoldX is calculated from a simple implementation of Coulomb's law, in which the dielectric constant scales with the burial of the bond under consideration 43. For calculations of ion–ion interactions, structural snapshots from MD simulations of PhCutA1 were taken every 20‐ for 400‐ns after an initial 100‐ns run (total, 16 structures).

## Conflict of interest

The authors declare no conflict of interest.

## Author contributions

KY and YJ designed the study; YM and KY performed the experiments; YM, BB, YJ, and KY analyzed the data; KY wrote the paper; all authors discussed the results and approved the final version for submission.

## Supporting information


**Table S1** A, 186 intra‐subunit interactions between favorable ion pairs in PhCutA1. B, 60 inter‐subunit interactions between favorable ion pairs in PhCutA1.
**Table S2.** Number of residues of PhCutA1 in each type of secondary structure in MD simulations (50–400 ns).
**Table S3 A**, Average distance between favorable intra‐subunit salt bridges in PhCutA1 during 400‐ns MD simulations using the indicated force fields. **B**, Average distance between favorable inter‐subunit salt bridges in PhCutA1 during 400‐ns MD simulations using the indicated force fields.
**Table S4.** Electrostatic energy of targeted residues for two structures from crystal analysis and six structures from MD simulation of PhCutA1.
**Table S5.** Comparison of percent occupancy of intra‐subunit salt bridges in PhCutA1 at each 100 ns during 400 ns MD simulation at 300 K using indicated force fields.
**Table S6.** Comparison of percent occupancy of intra‐subunit salt bridges in each subunit of PhCutA1 during 400 ns MD simulation at 300 K using indicated force fields.
**Table S7.** Side‐chain rotamer criteria of charged residues in PhCutA1.
**Table S8**. Buried ratio and pKa of negatively and positively charged residues in the crystal structure of PhCutA1.
**Table S9.** Buried ratio and pKa of negatively and positively charged residues in the crystal structure of PhCutA1.
**Table S10.** The number of Na^+^, Cl^−^, and H_2_O in the simulation box and size of the box during MD simulations at 300 K using indicated force fields.
**Fig. S1.** Comparison of helicity for PhCutA1 among six force fields in 300 K MD simulations (50–400 ns). (A) Percent helicity shows average values for each residue of PhCutA1. Red, Blue, and Black represent Charmm27_tip3p, Amber99sb_tip3p, and others, respectively. (B) Difference in helicity (Helicity subtraction of Amber99sb_tip3p from Charmm27_tip3p).
**Fig. S2.** Comparison of root‐mean‐square fluctuation (RMSF) for the C⍺ atoms of PhCutA1 among six force fields in 300 K MD simulations (50–400 ns). (A)Average RMSF values at each residue. (B)The difference values at each residue.
**Fig. S3.** Percent occupancy of salt bridges for each of targeted ionic pairs at six different force fields and the distance of salt bridges obtained from crystal structures. (A) Intra‐subunit interaction, (B) Inter‐subunit interaction.
**Fig. S4.** Snapshot of the configuration around Arg68 of PhCutA1.
**Fig. S5.** The configuration around Arg82 of PhCutA1. (A) The crystal structure of PhCutA1 (A, B, and C‐subunits of 4nyo). (B) The snapshot around Arg82 of PhCutA1 at 200 ns of an MD simulation in the case of Gromos53a6_spc/e.
**Fig. S6.** The snapshot of the configuration around N‐terminal (Met1) of PhCutA1 at 100 ns of an MD simulation using Amber99sb_tip3p.
**Fig. S7.** The snapshots of the configuration around Lys44 of PhCutA1 at 100 ns in an MD simulation using Charmm27_tip3p (A) and Gromos53a6_spc/e (B).
**Fig. S8.** The snapshots of the configuration around Lys70 of PhCutA1 at 100 ns in an MD simulation using Gromos43a1_spc/e (A) and Amber99sb_tip3p (B).
**Fig. S9.** The snapshots of the configuration around Arg36 of PhCutA1 at 200 ns in an MD simulation using Amber99sb_tip3p (A) and Gromos53a6_spc/e (B).
**Fig. S10.** The snapshots of the configuration around Lys101 and Lys102 of PhCutA1 at 100 ns in an MD simulation using Charmm27_tip3p (A) and Gromos53a6_spc/e (B).
**Fig. S11.** The snapshots of the configuration around Arg58 of PhCutA1 at 200 ns in an MD simulation using Amber14_tip3p (A) and Gromos43a1_spc/e (B).
**Fig. S12.** The snapshots of the configuration around Glu50 interacting with Lys56 of PhCutA1 at 100 ns in an MD simulation using Gromos43a1_spc/e (A) and Amber99sb_tip3p (B).
**Fig. S13.** The configuration around Asp84 and Asp86 of PhCutA1. (A) The crystal structure of PhCutA1 (A, B, and C‐subunits of 4nyo). (B) The snapshot of PhCutA1 at 200 ns of an MD simulation in the case of Gromos43a1_spc/e.
**Fig. S14.** The configuration around Arg33 of PhCutA1. (A) Snapshots around Arg33 and Glu34 of PhCutA1 at 100 ns of an MD simulation using Gromos43a1_spc/e. (B) Snapshots around Arg33 of PhCutA1 at 100 ns of MD simulation in the cases of Gromos43a1_spc/e (cyan) and Amber99sb_tip3p (yellow).
**Fig. S15.** Trajectories of distance between Arg33 in PhCutA1 and Cl^−^ ion during 400‐ns MD simulations at 300 K using indicated force fields. (A) Charmm27_tip3p, the distance between C_ζ_ of Arg33 in A‐subunit and Cl^−^ ion of the number 12197. The percent occupancy of distance (< 0.6 nm) between them was 100.0%. (B) Amber99sb_tip3p, the distance between C_ζ_ of Arg33 in A‐subunit and Cl^−^ ion of the number 12197. The percent occupancy of distance (< 0.6 nm) between them was 86.0%. (C) Gromos43a1_spc/e, the distance between C_ζ_ of Arg33 in C‐subunit and Cl^−^ ion of the number 12220. The percent occupancy of distance (< 0.6 nm) between them was 1.7%.
