# Supplementary material for: Evaluating the strengths of salt bridges in the CutA1 protein using molecular dynamic simulations: a comparison of different force fields
Source: FEBS Open Bio. 2019 Sep 27;9(11):1939–56. doi: 10.1002/2211-5463.12731 (PMC6823277; doi:10.1002/2211-5463.12731)

Supplemental Information

Evaluating the strengths of salt bridges in the CutA1 protein using molecular dynamics simulation: A comparison of different force fields

Yoshinori Matsuura[1], Yasumasa Joti[1,2], Bagautdin Bagautdinov[2], and Katsuhide Yutani[1*]

[1]RIKEN SPring-8 Center, 1-1-1 Kouto, Sayo, Hyogo 679-5148, Japan

[2]Japan Synchrotron Radiation Research Institute, 1-1-1, Kouto, Sayo, Hyogo 679-5198 Japan

Tables

Table S1A. 186 intra-subunit interactions between favorable ion pairs in PhCutA1

1	Met1AN_Glu59ACD	61	Lys44ACE_Glu47ACD	121	Lys70ACE_Asp76ACG
2	Met1BN_Glu59BCD	62	Lys44BCE_Glu47BCD	122	Lys70BCE_Asp76BCG
3	Met1CN_Glu59CCD	63	Lys44CCE_Glu47CCD	123	Lys70CCE_Asp76CCG
4	Met1AN_Asp84ACG	64	Lys49ACE_Asp10ACG	124	Arg82ACZ_Glu59ACD
5	Met1BN_Asp84BCG	65	Lys49BCE_Asp10BCG	125	Arg82BCZ_Glu59BCD
6	Met1CN_Asp84CCG	66	Lys49CCE_Asp10CCG	126	Arg82CCZ_Glu59CCD
7	Met1AN_Asp86ACG	67	Lys49ACE_Glu12ACD	127	Arg82ACZ_Asp60ACG
8	Met1BN_Asp86BCG	68	Lys49BCE_Glu12BCD	128	Arg82BCZ_Asp60BCG
9	Met1CN_Asp86CCG	69	Lys49CCE_Glu12CCD	129	Arg82CCZ_Asp60CCG
10	Lys16ACE_Asp10ACG	70	Lys49ACE_Glu34ACD	130	Arg82ACZ_Glu63ACD
11	Lys16BCE_Asp10BCG	71	Lys49BCE_Glu34BCD	131	Arg82BCZ_Glu63BCD
12	Lys16CCE_Asp10CCG	72	Lys49CCE_Glu34CCD	132	Arg82CCZ_Glu63CCD
13	Lys16ACE_Glu12ACD	73	Lys49ACE_Glu47ACD	133	Arg82ACZ_Asp84ACG
14	Lys16BCE_Glu12BCD	74	Lys49BCE_Glu47BCD	134	Arg82BCZ_Asp84BCG
15	Lys16CCE_Glu12CCD	75	Lys49CCE_Glu47CCD	135	Arg82CCZ_Asp84CCG
16	Lys16ACE_Glu15ACD	76	Lys49ACE_Asp48ACG	136	Lys94ACE_Glu90ACD
17	Lys16BCE_Glu15BCD	77	Lys49BCE_Asp48BCG	137	Lys94BCE_Glu90BCD
18	Lys16CCE_Glu15CCD	78	Lys49CCE_Asp48CCG	138	Lys94CCE_Glu90CCD
19	Lys19ACE_Glu12ACD	79	Arg58ACZ_Glu59ACD	139	Lys94ACE_Asp91ACG
20	Lys19BCE_Glu12BCD	80	Arg58BCZ_Glu59BCD	140	Lys94BCE_Asp91BCG
21	Lys19CCE_Glu12CCD	81	Arg58CCZ_Glu59CCD	141	Lys94CCE_Asp91CCG
22	Lys19ACE_Glu15ACD	82	Arg58ACZ_Asp60ACG	142	Lys94ACE_Glu98ACD
23	Lys19BCE_Glu15BCD	83	Arg58BCZ_Asp60BCG	143	Lys94BCE_Glu98BCD
24	Lys19CCE_Glu15CCD	84	Arg58CCZ_Asp60CCG	144	Lys94CCE_Glu98CCD
25	Lys23ACE_Glu24ACD	85	Arg58ACZ_Glu98ACD	145	Lys94ACE_Glu99ACD
26	Lys23BCE_Glu24BCD	86	Arg58BCZ_Glu98BCD	146	Lys94BCE_Glu99BCD
27	Lys23CCE_Glu24CCD	87	Arg58CCZ_Glu98CCD	147	Lys94CCE_Glu99CCD
28	Arg25ACZ_Glu24ACD	88	Arg58ACZ_Lys102AC	148	Lys101ACE_Glu24ACD
29	Arg25BCZ_Glu24BCD	89	Arg58BCZ_Lys102BC	149	Lys101BCE_Glu24BCD
30	Arg25CCZ_Glu24CCD	90	Arg58CCZ_Lys102CC	150	Lys101CCE_Glu24CCD
31	Arg25ACZ_Glu98ACD	91	Lys66ACE_Glu63ACD	151	Lys101ACE_Asp60ACG
32	Arg25BCZ_Glu98BCD	92	Lys66BCE_Glu63BCD	152	Lys101BCE_Asp60BCG
33	Arg25CCZ_Glu98CCD	93	Lys66CCE_Glu63CCD	153	Lys101CCE_Asp60CCG
34	Arg25ACZ_Glu99ACD	94	Lys66ACE_Glu67ACD	154	Lys101ACE_Glu64ACD
35	Arg25BCZ_Glu99BCD	95	Lys66BCE_Glu67BCD	155	Lys101BCE_Glu64BCD
36	Arg25CCZ_Glu99CCD	96	Lys66CCE_Glu67CCD	156	Lys101CCE_Glu64CCD
37	Arg25ACZ_Lys102AC	97	Arg68ACZ_Glu24ACD	157	Lys101ACE_Glu98ACD
38	Arg25BCZ_Lys102BC	98	Arg68BCZ_Glu24BCD	158	Lys101BCE_Glu98BCD
39	Arg25CCZ_Lys102CC	99	Arg68CCZ_Glu24CCD	159	Lys101CCE_Glu98CCD
40	Arg33ACZ_Glu34ACD	100	Arg68ACZ_Glu64ACD	160	Lys101ACE_Glu99ACD
41	Arg33BCZ_Glu34BCD	101	Arg68BCZ_Glu64BCD	161	Lys101BCE_Glu99BCD
42	Arg33CCZ_Glu34CCD	102	Arg68CCZ_Glu64CCD	162	Lys101CCE_Glu99CCD
43	Arg36ACZ_Glu34ACD	103	Arg68ACZ_Glu67ACD	163	Lys101ACE_Lys102AC
44	Arg36BCZ_Glu34BCD	104	Arg68BCZ_Glu67BCD	164	Lys101BCE_Lys102BC
45	Arg36CCZ_Glu34CCD	105	Arg68CCZ_Glu67CCD	165	Lys101CCE_Lys102CC
46	Arg36ACZ_Glu46ACD	106	Arg68ACZ_Glu71ACD	166	Lys102ACE_Glu24ACD
47	Arg36BCZ_Glu46BCD	107	Arg68BCZ_Glu71BCD	167	Lys102BCE_Glu24BCD
48	Arg36CCZ_Glu46CCD	108	Arg68CCZ_Glu71CCD	168	Lys102CCE_Glu24CCD
49	Arg36ACZ_Glu47ACD	109	Arg68ACZ_Lys102AC	169	Lys102ACE_Glu59ACD
50	Arg36BCZ_Glu47BCD	110	Arg68BCZ_Lys102BC	170	Lys102BCE_Glu59BCD
51	Arg36CCZ_Glu47CCD	111	Arg68CCZ_Lys102CC	171	Lys102CCE_Glu59CCD
52	Arg36ACZ_Asp48ACG	112	Lys70ACE_Glu63ACD	172	Lys102ACE_Asp60ACG
53	Arg36BCZ_Asp48BCG	113	Lys70BCE_Glu63BCD	173	Lys102BCE_Asp60BCG
54	Arg36CCZ_Asp48CCG	114	Lys70CCE_Glu63CCD	174	Lys102CCE_Asp60CCG
55	Lys44ACE_Glu42ACD	115	Lys70ACE_Glu67ACD	175	Lys102ACE_Glu63ACD
56	Lys44BCE_Glu42BCD	116	Lys70BCE_Glu67BCD	176	Lys102BCE_Glu63BCD
57	Lys44CCE_Glu42CCD	117	Lys70CCE_Glu67CCD	177	Lys102CCE_Glu63CCD
58	Lys44ACE_Glu46ACD	118	Lys70ACE_Glu71ACD	178	Lys102ACE_Glu64ACD
59	Lys44BCE_Glu46BCD	119	Lys70BCE_Glu71BCD	179	Lys102BCE_Glu64BCD
60	Lys44CCE_Glu46CCD	120	Lys70CCE_Glu71CCD	180	Lys102CCE_Glu64CCD
				181	Lys102ACE_Glu98ACD
				182	Lys102BCE_Glu98BCD
				183	Lys102CCE_Glu98CCD
				184	Lys102ACE_Glu99ACD
				185	Lys102BCE_Glu99BCD
				186	Lys102CCE_Glu99CCD

Table S1B. 60 inter-subunit interactions between favorable ion pairs in PhCutA1

1	Lys19ACE_Glu46CCD	21	Arg33CCZ_Glu47BCD	41	Lys66BCE_Asp91CCG
2	Lys19BCE_Glu46ACD	22	Arg36ACZ_Glu15BCD	42	Lys66CCE_Asp91ACG
3	Lys19CCE_Glu46BCD	23	Arg36BCZ_Glu15CCD	43	Lys70ACE_Glu90BCD
4	Lys19ACE_Glu47CCD	24	Arg36CCZ_Glu15ACD	44	Lys70BCE_Glu90CCD
5	Lys19BCE_Glu47ACD	25	Arg36ACZ_Glu34BCD	45	Lys70CCE_Glu90ACD
6	Lys19CCE_Glu47BCD	26	Arg36BCZ_Glu34CCD	46	Lys70ACE_Asp91BCG
7	Lys23ACE_Glu42CCD	27	Arg36CCZ_Glu34ACD	47	Lys70BCE_Asp91CCG
8	Lys23BCE_Glu42ACD	28	Lys44ACE_Glu15BCD	48	Lys70CCE_Asp91ACG
9	Lys23CCE_Glu42BCD	29	Lys44BCE_Glu15CCD	49	Arg82ACZ_Asp86BCG
10	Arg25ACZ_Glu42CCD	30	Lys44CCE_Glu15ACD	50	Arg82BCZ_Asp86CCG
11	Arg25BCZ_Glu42ACD	31	Lys56ACE_Glu50CCD	51	Arg82CCZ_Asp86ACG
12	Arg25CCZ_Glu42BCD	32	Lys56BCE_Glu50ACD	52	Arg82ACZ_Asp87BCG
13	Arg33ACZ_Glu34BCD	33	Lys56CCE_Glu50BCD	53	Arg82BCZ_Asp87CCG
14	Arg33ACZ_Glu34CCD	34	Lys66ACE_Asp87BCG	54	Arg82CCZ_Asp87ACG
15	Arg33BCZ_Glu34ACD	35	Lys66BCE_Asp87CCG	55	Lys101BCE_Glu42ACD
16	Arg33BCZ_Glu34CCD	36	Lys66CCE_Asp87ACG	56	Lys101CCE_Glu42BCD
17	Arg33CCZ_Glu34ACD	37	Lys66ACE_Glu90BCD	57	Lys101ACE_Glu42CCD
18	Arg33CCZ_Glu34BCD	38	Lys66BCE_Glu90CCD	58	Lys102ACE_Glu42CCD
19	Arg33ACZ_Glu47CCD	39	Lys66CCE_Glu90ACD	59	Lys102BCE_Glu42ACD
20	Arg33BCZ_Glu47ACD	40	Lys66ACE_Asp91BCG	60	Lys102CCE_Glu42BCD

Table S2. Number of residues of PhCutA1 in each type of secondary structure in MD simulations (50–400 ns)

force fields	structure*			β-sheet			α-helix		
Charmm27_tip3p	77.0	±	1.2	39.4	±	0.7	33.3	±	0.6
Amber99sb_spc/e	77.5	±	1.5	39.5	±	0.8	31.7	±	1.4
Amber99sb_tip3p	76.6	±	1.4	38.7	±	0.9	30.9	±	1.5
Amber14sp_tip3p	78.1	±	0.9	39.9	±	0.5	33.4	±	0.7
Gromos43a1_spc/e	76.6	±	2.0	38.0	±	1.7	33.6	±	0.5
Gromos53a6_spc/e	75.6	±	1.8	37.3	±	1.4	33.6	±	0.4

*structure = β-sheet + α-helix + β-bridge + turn

Values represent the average number of residues in each type of secondary structure among three subunits.

Table S3A. Average distance between favorable intra-subunit salt bridges in PhCutA1 during 400-ns MD simulations using the indicated force fields

targeted residues	interacting residues	Force fields					
		Charmm27_tip3p	Amber99sb_spc/e	Amber99sb_tip3p	Amber14sb_tip3p	Gromos43a1_spc/e	Gromos53a6_spc/e
N-term	Glu59	0.37 ± 0.08	0.44 ± 0.13	0.54 ± 0.17	0.37 ± 0.09	0.39 ± 0.07	0.38 ± 0.04
	Asp84	0.66 ± 0.05	0.62 ± 0.08	0.72 ± 0.17	0.72 ± 0.07	0.62 ± 0.06	0.67 ± 0.07
	Asp86	0.96 ± 0.05	0.98 ± 0.07	1.08 ± 0.14	0.99 ± 0.05	0.90 ± 0.09	0.70 ± 0.12
Lys16	Glu12	0.74 ± 0.19	0.75 ± 0.22	0.72 ± 0.21	0.70 ± 0.23	0.62 ± 0.18	0.64 ± 0.18
Lys19	Glu15	0.45 ± 0.10	0.45 ± 0.09	0.43 ± 0.07	0.45 ± 0.10	0.49 ± 0.08	0.49 ± 0.08
Arg25	Glu99	0.46 ± 0.06	0.48 ± 0.05	0.49 ± 0.07	0.49 ± 0.03	0.50 ± 0.11	0.48 ± 0.07
Arg33	Glu34	0.93 ± 0.04	1.09 ± 0.11	1.04 ± 0.09	0.91 ± 0.07	0.68 ± 0.20	0.83 ± 0.16
Arg36	Glu47	0.47 ± 0.09	0.53 ± 0.15	0.53 ± 0.14	0.64 ± 0.22	0.56 ± 0.12	0.56 ± 0.11
Lys44	Glu46	0.56 ± 0.15	0.63 ± 0.19	0.68 ± 0.20	0.72 ± 0.21	0.69 ± 0.23	0.59 ± 0.19
Lys49	Glu34	0.49 ± 0.15	0.47 ± 0.10	0.50 ± 0.14	0.61 ± 0.21	0.65 ± 0.19	0.58 ± 0.16
Arg58	Asp60	0.40 ± 0.02	0.42 ± 0.03	0.41 ± 0.02	0.41 ± 0.02	0.52 ± 0.11	0.48 ± 0.07
	C-term	0.62 ± 0.09	0.61 ± 0.07	0.62 ± 0.08	0.59 ± 0.08	0.79 ± 0.19	0.74 ± 0.16
Lys66	Glu63	0.58 ± 0.15	0.57 ± 0.18	0.51 ± 0.16	0.43 ± 0.11	0.55 ± 0.11	0.62 ± 0.11
	Glu67	0.78 ± 0.11	0.73 ± 0.16	0.82 ± 0.14	0.83 ± 0.14	0.70 ± 0.13	0.77 ± 0.13
Arg68	Glu24	0.41 ± 0.03	0.41 ± 0.03	0.43 ± 0.08	0.59 ± 0.18	0.58 ± 0.13	0.57 ± 0.12
	Glu71	0.52 ± 0.10	0.49 ± 0.08	0.49 ± 0.08	0.54 ± 0.14	0.65 ± 0.17	0.71 ± 0.16
Lys70	Glu67	0.71 ± 0.19	0.60 ± 0.20	0.64 ± 0.20	0.66 ± 0.19	0.63 ± 0.18	0.71 ± 0.17
Arg82	Glu59	0.64 ± 0.10	0.63 ± 0.13	0.61 ± 0.15	0.64 ± 0.11	0.61 ± 0.08	0.60 ± 0.07
Arg82	Asp84	0.40 ± 0.02	0.74 ± 0.07	0.77 ± 0.08	0.48 ± 0.07	0.67 ± 0.06	0.47 ± 0.08
Lys94	Glu90	0.77 ± 0.23	0.70 ± 0.24	0.70 ± 0.23	0.81 ± 0.23	0.69 ± 0.20	0.77 ± 0.20
	Asp91	0.68 ± 0.13	0.67 ± 0.13	0.63 ± 0.15	0.69 ± 0.15	0.67 ± 0.15	0.72 ± 0.14
Lys101	Glu64	0.47 ± 0.10	0.46 ± 0.10	0.49 ± 0.12	0.70 ± 0.12	1.20 ± 0.34	1.27 ± 0.32
	C-term	0.62 ± 0.07	0.60 ± 0.07	0.61 ± 0.08	0.59 ± 0.07	0.75 ± 0.10	0.76 ± 0.09
Lys102	Glu98	0.66 ± 0.27	0.68 ± 0.26	0.70 ± 0.28	0.61 ± 0.23	1.27 ± 0.44	1.43 ± 0.37

These values represent the average of three subunits.
All distances are in nm.
These data are shown when ion-pairs less than 0.7 nm were detected.at least once among six force fields.
Yellow and orange represent, respectively, the lowest and highest values of the distance among six force fields.

Table S3B. Average distance between favorable inter-subunit salt bridges in PhCutA1 during 400-ns MD simulations using the indicated force fields

targeted residues		interacting residues		Force fields					
				Charmm27_tip3p	Amber99sb_spc/e	Ambeer99sb_tip3p	Amber14sb_tip3p	Gromos43a1_spc/e	Gromos63a6_spc/e
Lys19	A	Glu47	C	0.62 ± 0.24	0.58 ± 0.20	0.47 ± 0.09	0.61 ± 0.17	0.70 ± 0.19	0.69 ± 0.16
	B		A	0.50 ± 0.13	0.51 ± 0.12	0.46 ± 0.07	0.57 ± 0.15	0.61 ± 0.18	0.65 ± 0.17
	C		B	0.46 ± 0.10	0.50 ± 0.12	0.46 ± 0.07	0.57 ± 0.16	0.69 ± 0.16	0.67 ± 0.17
Arg33	A	Glu34	C	1.24 ± 0.07	1.12 ± 0.18	1.22 ± 0.12	1.20 ± 0.14	0.65 ± 0.14	0.98 ± 0.16
	B		A	1.22 ± 0.07	1.26 ± 0.17	1.26 ± 0.10	1.21 ± 0.11	0.52 ± 0.09	0.71 ± 0.25
	C		B	1.15 ± 0.11	1.05 ± 0.20	0.96 ± 0.23	1.19 ± 0.14	0.58 ± 0.16	0.59 ± 0.11
Arg36	A	Glu15	B	0.66 ± 0.13	0.60 ± 0.18	0.59 ± 0.17	0.62 ± 0.16	0.66 ± 0.15	0.75 ± 0.14
	B		C	0.63 ± 0.14	0.59 ± 0.18	0.59 ± 0.18	0.70 ± 0.14	0.66 ± 0.15	0.75 ± 0.15
	C		A	0.63 ± 0.15	0.60 ± 0.18	0.47 ± 0.11	0.63 ± 0.16	0.68 ± 0.15	0.74 ± 0.14
Lys56	A	Glu50	C	0.76 ± 0.07	0.69 ± 0.11	0.90 ± 0.12	0.85 ± 0.09	0.63 ± 0.08	0.66 ± 0.05
	B		A	0.76 ± 0.06	0.67 ± 0.08	0.81 ± 0.12	0.83 ± 0.10	0.53 ± 0.05	0.66 ± 0.07
	C		B	0.73 ± 0.05	0.68 ± 0.09	0.85 ± 0.11	0.85 ± 0.10	0.60 ± 0.07	0.67 ± 0.07
Lys66	A	Asp87	B	0.61 ± 0.08	0.59 ± 0.11	0.60 ± 0.11	0.55 ± 0.07	0.61 ± 0.07	0.71 ± 0.10
	B		C	0.62 ± 0.06	0.60 ± 0.10	0.54 ± 0.09	0.57 ± 0.07	0.64 ± 0.06	0.70 ± 0.10
	C		A	0.62 ± 0.07	0.59 ± 0.09	0.59 ± 0.09	0.58 ± 0.07	0.63 ± 0.07	0.69 ± 0.10
	A	Glu90	B	0.77 ± 0.24	0.86 ± 0.20	1.13 ± 0.16	0.72 ± 0.16	0.69 ± 0.18	0.68 ± 0.18
	B		C	0.60 ± 0.22	0.94 ± 0.22	1.02 ± 0.29	0.71 ± 0.15	0.72 ± 0.18	0.66 ± 0.18
	C		A	0.66 ± 0.25	0.79 ± 0.20	0.73 ± 0.23	0.69 ± 0.16	0.69 ± 0.18	0.64 ± 0.17
Lys70	A	Asp91	B	0.61 ± 0.14	0.84 ± 0.21	1.04 ± 0.18	0.65 ± 0.13	0.63 ± 0.17	0.58 ± 0.13
	B		C	0.54 ± 0.12	0.98 ± 0.21	0.89 ± 0.25	0.59 ± 0.11	0.55 ± 0.13	0.58 ± 0.13
	C		A	0.56 ± 0.13	0.78 ± 0.22	0.59 ± 0.18	0.61 ± 0.12	0.53 ± 0.14	0.57 ± 0.14
Arg82	A	Asp86	B	0.64 ± 0.09	0.45 ± 0.05	0.44 ± 0.04	0.59 ± 0.05	0.50 ± 0.08	0.84 ± 0.13
	B		C	0.63 ± 0.05	0.45 ± 0.05	0.44 ± 0.03	0.61 ± 0.05	0.51 ± 0.08	0.83 ± 0.13
	C		A	0.63 ± 0.05	0.44 ± 0.03	0.44 ± 0.03	0.50 ± 0.10	0.52 ± 0.11	0.82 ± 0.14
	A	Asp87	B	0.62 ± 0.05	0.55 ± 0.08	0.60 ± 0.11	0.60 ± 0.06	0.62 ± 0.07	0.50 ± 0.07
	B		C	0.62 ± 0.05	0.54 ± 0.08	0.54 ± 0.09	0.59 ± 0.06	0.60 ± 0.07	0.50 ± 0.07
	C		A	0.62 ± 0.05	0.52 ± 0.08	0.51 ± 0.07	0.57 ± 0.08	0.60 ± 0.08	0.51 ± 0.07

All distances are in nm.
A, B, and C represent the A-, B-, C-subunits of PhCutA1, respectively
These data are shown when ion-pairs less than 0.7 nm were detected.at least once among six force fields.
Yellow and orange represent, respectively, the lowest and highest values of the distance among six force fields.

Table S4. Electrostatic energy of targeted residues for two structures from crystal analysis and six structures from MD sinulation of PhCutA1

Targeted sites	Mutations	ΔTd (°C)*	Elecrostatic energy of targeted residues (kJ/mol)							
			Crystal structures of 4nyo		Structures of MD simulations					
			ABC	DEF	Charmm27 _tip3p	Amber99sb _spc/e	Amber99sb _tip3p	Amber14sb _tip3p	Gromos43a1 _spc/e	Gromos53a6 _spc/e
D48	D48N	2.7	10.5	10.8	8.2	6.1	7.6	10.4	8.8	7.5
D60	D60A	-3.3	-8.8	-7.3	-3.3	-6.9	-8.1	-6.1	-3.2	-5.0
D76	D76N	0.9	3.3	3.1	3.3	1.8	2.5	4.1	4.8	3.6
D84	D84A	-1.5	7.2	6.8	4.1	11.1	11.5	9.0	10.2	-2.1
D86	D86N	2.5	19.6	19.0	16.5	4.5	4.9	15.2	6.5	7.0
D87	D87N	-7.0	1.5	2.2	2.3	-1.8	-1.3	1.1	5.4	3.0
D91	D91A	-6.6	-3.5	0.5	-0.4	3.6	1.5	1.9	-1.3	-1.0
E12	E12Q	0.7	11.6	10.8	7.9	6.5	6.4	7.7	8.2	9.7
E15	E15A	-0.8	3.8	1.0	0.0	-2.8	-3.1	-1.4	-0.2	1.1
E24	E24A	-3.0	-15.6	-15.9	-15.1	-15.3	-14.5	-9.0	-6.2	-5.3
E34	E34Q	-1.4	-11.0	-14.4	-10.3	-8.7	-9.1	-10.9	-18.2	-16.2
E42	E42Q	4.9	2.5	3.7	1.8	1.0	1.8	2.4	-0.9	-0.1
E46	E46Q	3.6	5.5	6.2	3.3	1.9	3.0	5.8	3.4	1.4
E47	E47A	-2.5	3.1	0.8	0.3	-2.5	-1.1	1.6	-1.8	-1.4
E59	E59Q	3.7	-7.3	-6.6	-4.7	-2.3	-2.6	-4.5	-5.0	-4.3
E63	E63A	0.5	5.1	5.5	7.6	5.5	6.3	5.8	4.7	5.8
E64	E64A	-2.9	-0.8	3.2	4.7	2.1	3.5	3.3	-0.2	1.9
E67	E67A	0.5	5.2	5.4	5.5	3.5	3.7	0.3	3.0	3.5
E71	E71A	-0.5	-5.7	-4.4	-4.6	-5.5	-6.1	-5.0	-1.1	-0.6
E99	E99A	1.5	-9.1	-5.1	-7.3	-7.4	-6.4	-7.6	-3.8	-6.9
K101	K101A	-4.4	-6.7	-4.5	-7.1	-8.6	-7.6	-5.9	-3.9	-3.8
K19	K19A	-2.6	-3.6	-6.5	-7.2	-8.5	-10.4	-5.7	-6.2	-6.1
K49	K49A	1.0	-10.2	-8.6	-8.9	-10.3	-10.3	-6.0	-8.3	-7.4
K66	K66A	-6.5	-21.5	-18.7	-16.1	-15.0	-13.4	-14.2	-14.1	-12.3
K70	K70A	-3.4	-16.2	-10.8	-10.1	-7.7	-7.3	-8.9	-10.1	-10.4
R25	R25A	-12.4	-12.0	-6.9	-10.1	-10.4	-9.5	-9.6	-12.0	-12.2
R33	R33A	-9.2	10.9	3.5	5.8	2.3	3.9	1.6	-4.9	-0.6
R36	R36A	-1.9	-13.5	-11.9	-18.8	-22.9	-22.4	-17.3	-14.7	-14.1
R58	R58A	-6.8	-21.8	-25.2	-21.7	-22.5	-22.7	-22.9	-14.9	-16.0
R68	R68A	-2.1	-28.0	-26.0	-25.2	-27.4	-26.7	-31.3	-23.7	-15.3
R82	R82A	-10.0	-35.7	-34.8	-34.2	-32.6	-34.5	-34.6	-31.6	-35.5

*Difference in denaturation temperatures of PhCutA1 mutants (17).

Table S5. Comparison of percent occupancy of intra-subunit salt bridges in PhCutA1 at each 100-ns during 400-ns MD simulation at 300 K using indicated force fields

	Force fields					
	Charmm27 _tip3p	Amber99sb _spc/e	Amber99sb _tip3p	Amber14sb _tip3p	Gromos43a1 _spc/e	Gromos53a6 _spc/e
0-100 ns	81.5	102.6	76.0	77.2	84.1	73.2
100-200 ns	83.5	83.5	78.3	77.6	74.2	74.2
200-300 ns	83.8	74.9	81.6	74.4	77.6	74.9
300-400 ns	81.3	73.6	83.1	81.6	80.3	73.6
Average(1)	82.5 ± 1.3	83.6 ± 13.4	79.7 ± 3.2	77.7 ± 3.0	79.0 ± 4.2	74.0 ± 0.7
Average(2)	82.9 ± 1.4	77.3 ± 5.4	81.0 ± 2.4	77.9 ± 3.6	77.4 ± 3.0	74.2 ± 0.6

Data show average values of percent occupancies of 17 positively charged residues indicated in Table 2A.

Average(1) and (2) represent average values from 0 to 400-ns and 100 to 400-ns, respectively.

Table S6. Comparison of percent occupancy of intra-subunit salt bridges in each subunit of PhCutA1 during 400-ns MD simulation at 300 K using indicated force fields

	Force fields					
	Charmm27 _tip3p	Amber99sb _spc/e	Amber99sb _tip3p	Amber14sb _tip3p	Gromos43a1 _spc/e	Gromos53a6 _spc/e
A-subunit	82.9	84.3	78.4	79.0	79.9	71.8
B-subunit	82.4	82.6	79.2	78.9	78.7	74.8
C-subunit	82.3	84.0	81.6	75.3	78.6	75.4
average	82.5	83.6	79.7	77.7	79.0	74.0
STDEV	0.3	0.9	1.6	2.1	0.7	1.9

Data show average values of percent occupancies of 17 positively charged residues indicated in Table 2A.

STDEV represent the the standard deviation of average values for 3 subunits.

Table S7. Side-chain rotamer criteria of charged residues in PhCutA1

The side chains of charged residues in PhCutA1 shown in Figure S4 to Figure S14 were examined by MolProbity (http://molprobity.biochem.duke.edu).

(p (plus, centered near +60°), t (trans, centered near 180°), and m (minus, centered near -60°))

Figure	Force field	Residue	Rotamer's configuration	Validation (Favored (>2.0%), Allowed (0.3 - 2.0%), Outlier (≤0.3%))	Dihedral angles, chi1, chi2, chi3, chi4
S4	Amber99sb_tip3p (at 100-ns)	Arg68B	tpt170	25.9	178.9, 56.9, 186.7, 149.9
		Glu64B	tt0	26.9	189.6, 183.6, 117.2
		Glu24B	tt0	1.1	153.9, 163.7, 249.5
		Glu71B	mt-10	8.5	314.0, 193.3, 298.6
	Amber14sb_tip3p (at 100-ns)	Arg68B	mmm-85	34	293.2, 291.5, 282.5, 287.1
		Glu64B	tp30	30.6	185.6, 66.4, 177.1
		Glu24B	mm-30	33.8	289.8, 282.7, 320.3
		Glu71B	mm-30	77.9	293.9, 307.4, 136.7
S5	4nyo (crystal structure)	Arg82A	ttt180	35.5	164.0, 181.5, 169.0, 164.9
		Asp86B	m-30	36	297.3, 287
		Asp87B	t0	30.6	198.4, 196.4
		Glu63A	mm-30	77.4	293.8, 302.9, 327.9
		Asp84A	m-30	18.8	293.2, 187.8
		Glu59A	mt-10	36.1	289.5, 166.8, 43.7
	Gromos53a6_spc/e (at 200-ns)	Arg82A	ttt180	11	183, 189.5, 160.4, 145.8
		Asp86B	m-30	5.8	275.3, 128.7
		Asp87B	m-30	2.5	290.6, 29.3
		Glu63A	mt-10	50.3	294.7, 177.6, 228.1
		Asp84A	t70	33.5	188.6, 57.8
		Glu59A	pm20	20.1	63.7, 282.7, 8.5
S6	Amber99sb_tip3p (at 100-ns)	Met1	mtt	20.8	282.8, 199.3, 182.4
		Glu59	tt0	38	186.2, 171.1, 243.5
		Asp84	tt0	9	171.7, 55.7
		ASP86	T70	32.7	180.9,239.1
S7	Charmm27_tip3p (at 100-ns)	Lys44A	pttp	3.4	62.6, 189.4, 165.7, 35.5
		Glu42A	tt0	8.3	201, 164.1, 243.1
		Glu46A	mm-30	7.9	270.1, 296.6, 329.8
	Gromos53a6_spc/e (at 100-ns)	Lys44A	mtmt	45.7	297.3, 183.4, 301.6, 183.9
		Glu42A	mt-10	13.8	298.6, 200.3, 82.8
		Glu46A	mm-30	41.6	295.6,283.4,324.5
S8	Gromos43a1_spc/e (at 100-ns)	Lys70B	pttt	44.8	69.4,176.7,179.2,166.7
		Glu67B	mt-10	30	303.6, 186.3, 233.4
		Asp76B	p0	1.1	45.1 106.1
		Glu90C	mp0	1.7	288.6, 60, 258
		Asp91C	m-30	6.1	300, 263.5
	Amber99sb_tip3p (at 100-ns)	Lys70B	mtmt	9.4	283.3,178, 307.6, 208.5
		Glu67B	tp30	4.7	200, 59.1,77.1
		Asp76B	t70	18	193.6, 250.2
		Glu90C	mt-10	35.1	287.6, 185.4, 99.3
		Asp91C	t70	27.6	191.7, 242.4
S9	Amber99sb_tip3p (at 200-ns)	Arg36A	mtp180	81.3	294,186,62.1,190.5
		Glu34A	tt0	23.1	172.9, 187.5, 76.9
		Glu47A	outlier	0.1	58.2,257.7, 296.3
		Glu15B	mt-10	38.7	292.4, 183.4, 78.5
		Glu34B	tt0	11.5	190.1,193.8, 98.5
	Gromos53a6_spc/e (at 200-ns)	Arg36A	mtt180	49.9	289.6, 191.2, 191.4,155.1
		Glu34A	tt0	8.4	197.9,193.6,62.2
		Glu47A	pm20	1.4	51.4, 279.5, 64.3
		Glu15B	tt0	31.5	187.6, 176.6, 266.3
		Glu34B	mp0	1.5	314.9, 82.8, 121.2

Table S7 Continued

S10	Charmm27_tip3p	Lys101B	pttt	27.9	73.9, 162.8, 190, 177.4
	(at 100-ns)	Lys102B	mmtm	18.3	305.7, 306.1, 183.4, 314.5
		Glu64B	tt0	37.4	174.5, 165.2, 25.7
		Glu98B	mm-30	25	298.4, 285.2, 303.8
	Gromos53a6_spc/e	Lys101B	pttt	37.9	50.2, 190.9, 176.9, 185.5
	(at 100-ns)	Lys102B	tttt	18.4	205.7, 196.5, 175.5, 171.3
		Glu64B	mt-10	0.3	253.7, 165.3, 268.4
		Glu98B	mm-30	18.2	289.9, 279.2, 131.8
S11	Amber14sb_tip3p	Arg58A	mtm-85	4.7	310.2, 194.1, 311.7, 232.2
	(at 200-ns)	Asp60A	p0	2.5	75.6, 303.3
		Lys102A	mtpp	5.9	308.8, 201.9, 72.7, 69.9
	Gromos43a1_spc/e	Arg58A	ptt180	22.6	57.6, 174.9, 165.3, 169.7
	(at 200-ns)	Asp60A	m-30	5.4	299.4, 80.5
		Lys102A	pttp	10	76.3, 194.4, 175.5, 55.1
S12	Gromos43a1_spc/e	Glu50A	mt-10	34.3	281.9, 187,134.4
	(at 100-ns)	Lys56B	mttp	3.1	311, 191.1,180.5, 118.8
	Amber99sb_tip3p	Glu50A	tt0	33.5	187.5, 186.6, 230
	(at 100-ns)	Lys56B	mttt	90.6	302.5, 177, 181.7, 176.7
S13	4nyo	Arg82A	ttt180	35.5	164, 181.5, 169, 164.9
	(crystal structure)	Asp84A	m-30	18.8	293.2, 187.8
		Asp86A	m-30	18.7	297.1, 277.6
		Arg82B	ttt180	49.9	166.8, 187.2, 171.3, 177.8
		Asp84B	m-30	6.6	287.1, 21.2
		Asp86B	m-30	36	297.3, 287
		Arg82C	ttt180	56.5	168.6, 182.1, 174.3, 169.4
		Asp84C	m-30	3.4	282.7, 29.3
		Asp86C	m-30	31.8	296.6, 285.2
	Gromos43a1_spc/e	Arg82A	ttt180	11	183, 189.5, 160.4, 145.8
	(at 200-ns)	Asp84A	t70	33.5	188.6, 57.8
		Asp86A	t0	22.5	198.2, 34.2
		Arg82B	ttt180	18.1	185.9, 177.3, 153.3, 159.2
		Asp84B	m-30	19	290.6, 103.1
		Asp86B	m-30	5.8	275.3, 128.7
		Arg82C	ttt180	24.1	171.7, 181.1, 166, 151.2
		Asp84C	m-30	1.8	283.1, 263.9
		Asp86C	t70	7.6	197.7, 261.3
S14	Gromos43a1_spc/e	Arg33A	ttt-90	1.6	205.7, 145.5, 189.3, 235.2
	(at 100-ns)	Glu34A	pm20	0.3	53.5, 286.5, 81.3
		Arg33B	tpt-90	1.8	207.5, 79.6, 189.9, 243.5
		Glu34B	pm20	0.9	76, 266.6, 69.5
		Arg33C	tpt-90	4.7	195, 180.4, 151.8, 232.2
		Glu34C	pm20	0.9	223.6, 176.6, 107.2
	Amber99sb_tip3p	Arg33A	ptt90	53.5	68.7, 171.7, 176.9, 85.3
	(at 100-ns)	Glu34A	tt0	23.4	192.7, 175.3, 296.8
		Arg33B	ptt-90	21.6	71.5, 164, 184.1, 266.5
		Glu34B	tt0	1.1	153.9, 163.7, 249.5
		Arg33C	pmt-80	1.6	83.4, 302.1, 177.8, 293
		Glu34C	tt0	4.7	209.6, 173.4, 130.3

Table S8. Percent occupancies of salt bridges (less than 0.6 nm) of negatively charged residues in PhCutA1 with Na^+ ions during 400-ns MD simulations at 300 K using the indicated force fields

Negatively charged Residues	Force fields											
	Charmm27 _tip3p		Amber99sb _spc/e		Amber99sb _tip3p		Amber14sb _tip3p		Gromos43a1 _spc/e		Gromos53a6 _spc/e	
	P. C. Residue*	Na^+ ion	P. C. Residue*	Na^+ ion	P. C. Residue*	Na^+ ion	P. C. Residue*	Na^+ ion	P. C. Residue*	Na^+ ion	P. C. Residue*	Na^+ ion
Asp10	5.0	32.6	4.4	13.5	6.0	22.0	0.4	40.0	7.8	10.1	0.8	12.8
Glu12	28.6	50.3	29.4	23.1	32.9	23.1	40.3	67.2	54.2	16.8	47.8	18.8
Glu15	125.7	67.3	148.4	14.7	162.3	16.9	127.4	78.2	125.2	10.9	104.1	10.2
Glu24	114.5	5.7	111.2	3.2	109.5	4.1	76.7	16.5	73.0	12.8	80.2	9.3
Glu34	82.7	11.0	94.2	4.8	88.3	5.0	57.4	12.8	175.5	1.2	140.2	1.6
Glu42	19.6	37.0	12.2	20.6	14.4	19.4	8.3	48.3	8.8	17.9	3.2	19.4
Glu46	64.9	61.5	49.9	27.8	40.4	24.2	32.2	107.0	42.1	26.3	60.5	27.8
Glu47	162.9	68.1	144.1	18.9	171.3	20.2	114.6	102.4	111.7	13.5	106.6	12.5
Asp48	10.7	87.8	18.9	47.1	8.2	54.0	4.7	114.7	9.2	36.8	2.5	35.5
Glu50	0.2	2.4	12.6	2.3	0.3	4.3	0.0	14.7	59.7	7.9	13.9	2.5
Glu59	28.0	23.6	46.1	28.9	50.2	19.4	46.0	19.6	57.2	23.7	60.3	14.2
Asp60	100.4	21.0	98.8	12.9	100.1	11.4	99.9	24.5	121.1	12.9	123.8	8.7
Glu63	52.3	36.0	60.0	22.2	72.3	19.1	89.0	53.3	72.8	23.6	44.9	22.8
Glu64	95.9	30.0	91.1	22.5	83.7	24.5	51.9	72.9	91.2	17.6	80.2	18.7
Glu67	39.1	50.6	79.4	23.6	59.4	25.2	93.7	81.9	83.8	19.6	43.9	24.9
Glu71	86.3	47.6	93.3	18.2	95.0	18.7	75.1	65.5	50.7	19.7	30.1	21.8
Asp76	0.3	50.0	18.1	40.0	15.9	35.4	0.5	59.4	0.9	43.7	0.4	28.2
Asp84	113.1	87.8	47.2	104.3	42.8	87.4	83.4	125.5	44.3	79.2	101.9	46.1
Asp86	25.5	159.4	98.0	74.9	99.8	56.7	57.9	153.2	91.6	63.1	21.9	63.6
Asp87	49.6	17.8	117.7	14.6	121.9	13.1	109.4	49.3	65.3	22.4	99.4	22.0
Glu90	74.3	28.2	53.9	15.8	56.9	17.3	56.2	63.0	70.4	11.7	62.7	9.7
Asp91	83.1	12.2	39.9	16.1	64.4	17.2	69.6	32.5	93.6	11.1	77.6	8.1
Glu98	58.7	27.7	57.8	16.1	49.1	19.4	66.1	38.7	64.3	16.0	32.8	18.9
Glu99	97.2	9.5	96.1	6.4	91.3	7.5	99.3	16.1	97.5	12.1	106.3	12.4
C-terminal	71.2	45.8	87.9	17.8	76.6	18.1	105.5	33.7	29.3	17.3	25.4	13.8
average	63.3	42.8	67.6	24.4	68.2	23.4	60.8	59.6	69.7	21.9	60.2	19.4

*P. C. Residue represents percent occupancies with Positively Charged Residues. Data come from Table 2A and 2B.

Table S8. Buried ratio and pKa of negatively and positively charged residues in the crystal structure of PhCutA1*

Residues	Buried ratio			pKa		
	A-subunit	B-subunit	C-subunit	A-subunit	B-subunit	C-subunit
ASP 10	0.55	0.25	0.84	3.58	3.13	2.7
ASP 48	0.58	0.51	0.9	5.12	4.53	8.26
ASP 60	0.02	0.07	0.06	2.07	2.43	2.17
ASP 76	0	0	0	4.04	4.01	3.97
ASP 84	0.28	0.31	0.3	3.01	3.06	3.31
ASP 86	0.36	0.39	0.41	4.36	4.59	4.94
ASP 87	0.23	0.24	0.23	3.23	3.21	3.43
ASP 91	0.26	0.36	0.34	3.87	3.27	3.09
GLU 12	0.42	0	0.49	3.8	4.77	4.7
GLU 15	1	0.38	0.93	11.09	5.06	1.42
GLU 24	0.04	0.07	0.06	3.07	3.84	3.25
GLU 34	1	1	1	5.77	7.6	7.27
GLU 42	0	0	0	4.73	4.45	4.45
GLU 46	0	0	0.5	4.67	3.67	3.58
GLU 47	0.33	0.67	1	4.48	5.57	6.37
GLU 50	1	1	1	6.78	6.46	6.23
GLU 59	0.16	0.06	0.2	3.91	5.22	3.9
GLU 63	0.01	0	0	3.89	3.92	4.33
GLU 64	0	0.03	0.04	3.54	3.74	3.93
GLU 67	0	0	0	4.54	4.78	3.36
GLU 71	0	0	0	4.06	4.21	4.28
GLU 90	0	0	0	4.48	4.75	4.89
GLU 98	0	0	0	4.67	3.89	3.43
GLU 99	0.35	0.28	0.26	5.76	4.85	4.72
C- 102	0	0	0	3.21	2.87	2.77
LYS 16	0	0	0.12	10.57	10.41	11.49
LYS 19	0.62	0.06	0.57	11.71	10.64	10.88
LYS 23	0	0	0	10.32	10.2	10.43
LYS 44	0	0	0	10.62	11.31	10.62
LYS 49	0.33	0.74	1	10.62	10.32	12.05
LYS 56	1	1	1	7.3	7.7	7.8
LYS 66	0.14	0.2	0.2	11.74	10.67	12.01
LYS 70	0.07	0.18	0.07	11	11.56	11.06
LYS 94	0	0	0	10.66	11.06	11.49
LYS 101	0	0	0	11.33	11.5	11.48
LYS 102	0	0.01	0	10.4	10	10.48
ARG 25	0.13	0.14	0.14	12.19	12.61	12.53
ARG 33	1	1	1	13.57	14.68	12.39
ARG 36	0.69	1	1	12.57	12.1	14.23
ARG 58	0.15	0.18	0.17	13.81	13.65	13.37
ARG 68	0	0	0	14.78	14.07	14.61
ARG 82	0.27	0.27	0.27	13.65	13.81	13.43

*These values in the crystal structure (4nyo) were estimated using a software, propka3.0, revision 182 in http://nbcr-222.ucsd.edu/pdb2pqr_2.0.0/

Table S10. The number of Na^+, Cl^-, and H_2O in the simulation box and size of the box during MD simulations at 300 K using indicated force fields

	Charmm27 _tip3p	Amber99sb _spc/e	Amber99sb _tip3p	Amber14sb _tip3p	Gromos43a1 _spc/e	Gromos53a6 _spc/e
Na^+	59	59	58	59	59	59
Cl^-	38	39	38	38	38	38
H_2O	12011	12011	12011	12011	12023	12022
Box-X*	8.10715	8.07810	8.10875	8.10591	8.07218	8.09331
Box-Y*	7.64355	7.61617	7.64507	7.64238	7.61058	7.63050
Box-Z*	6.61970	6.59599	6.62102	6.61869	6.59115	6.60840

*The average values (nm) of simulation box during 400 ns at 300 K

Figures

Figure S1. Comparison of helicity for PhCutA1 among six force fields in 300 K MD simulations (50–400 ns)

(A) Percent helicity shows average values for each residue of PhCutA1. Red, Blue, and Black represent Charmm27_tip3p, Amber99sb_tip3p, and others, respectively.

(B) Difference in helicity (Helicity subtraction of Amber99sb_tip3p from Charmm27_tip3p)

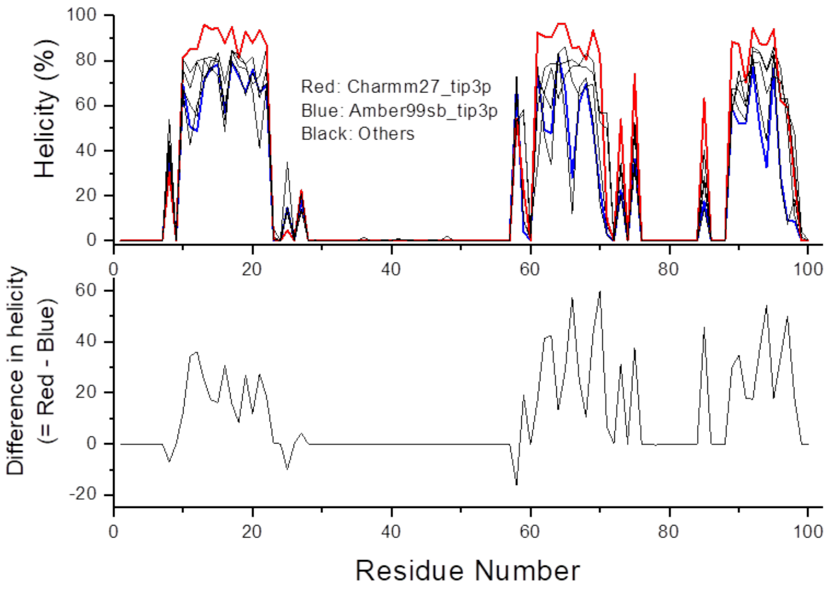

Figure S2. Comparison of root-mean-square fluctuation (RMSF) for the C_{α} atoms of PhCutA1 among six force fields in 300 K MD simulations (50–400 ns)

(a), (b), (c), (d), (e), and (f) represent Charmm27_tip3p, Amber99sb_spc/e, Amber99sb_tip3p, Amber14sb_tip3p, Gromos43a1_spc/e, and Gromo53a6_spc/e, respectively.

(A) Average RMSF values at each residue

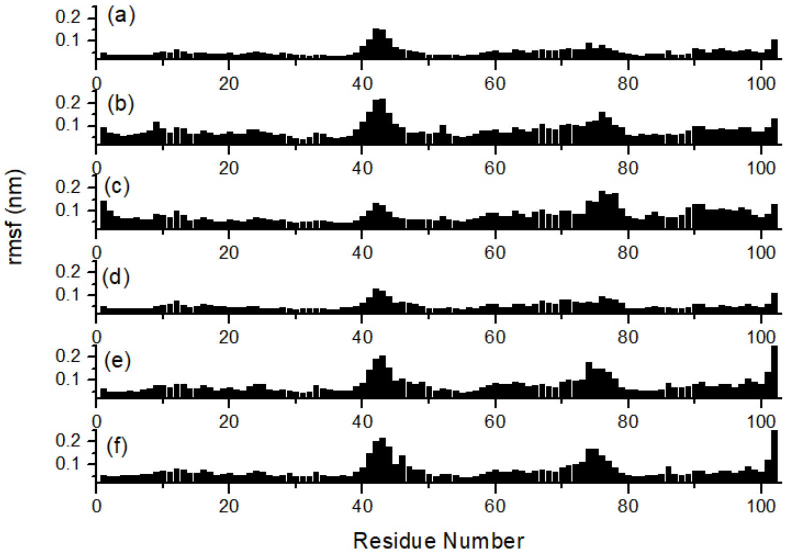

(B) The difference values at each residue

The differences in average RMSF at each Cα atom were obtained by subtracting the average value of Cα atoms in six force fields from a value of each Cα atom.

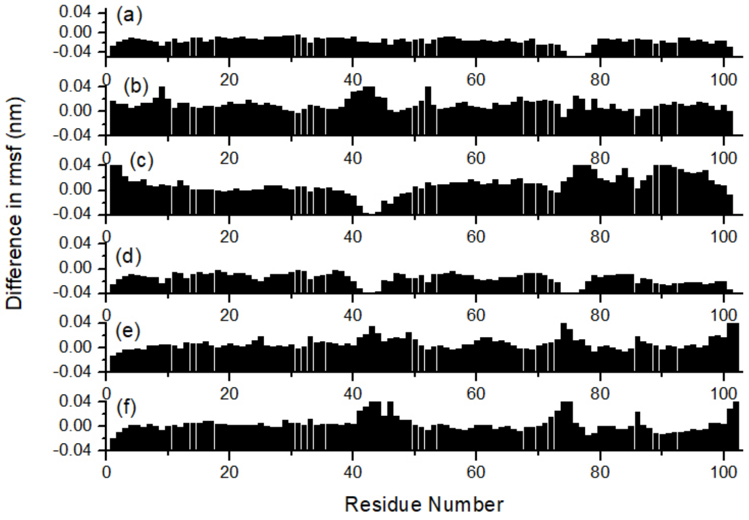

Figure S3. Percent occupancy of salt bridges for each of targeted ionic pairs at six different force fields and the distance of salt bridges obtained from crystal structures.

(A) Intra-subunit interaction, (B) Inter-subunit interaction.

Targeted ionic pairs are listed in Tables S1A and S1B for intra- and inter-subunit interaction, respectively. Three bars represent the data for each targeted pair in A, B, and C-subunits. The lengths of salt bridges were calculated by the CCP4 software using the structures of ABC and DEF for PDB ID 4nyo and AB for 1umj. B-factors of charged residues in crystal analysis are indicated by a color gradient: blue indicates the lowest B-factors and red the highest. The upper line, with the color gradient showing B-factors, represents the B-factors of negatively charged residues; the other one is positively charged.

(a), (b), (c), (d), (e), and (f) represent Charmm27_tip3p, Amber99sb_spc/e, Amber99sb_tip3p, Amber14sb_tip3p, Gromos43a1_spc/e, and Gromos53a6_spc/e, respectively.

Fig. S3A

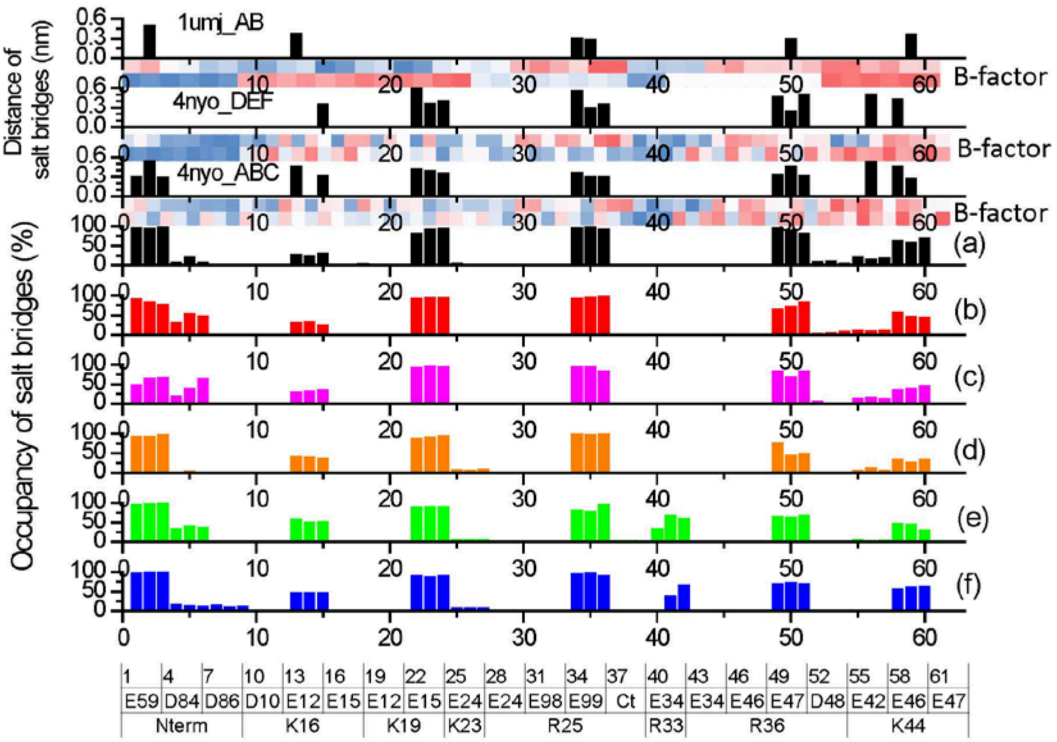

Fig. S3A continued

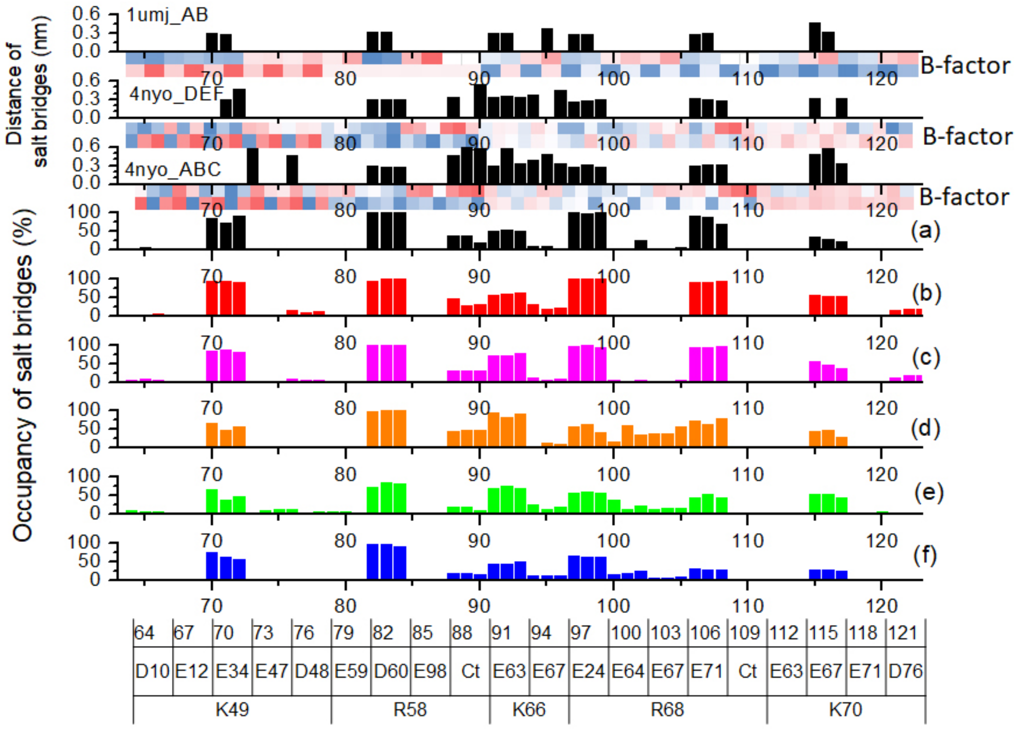

Fig. S3A continued

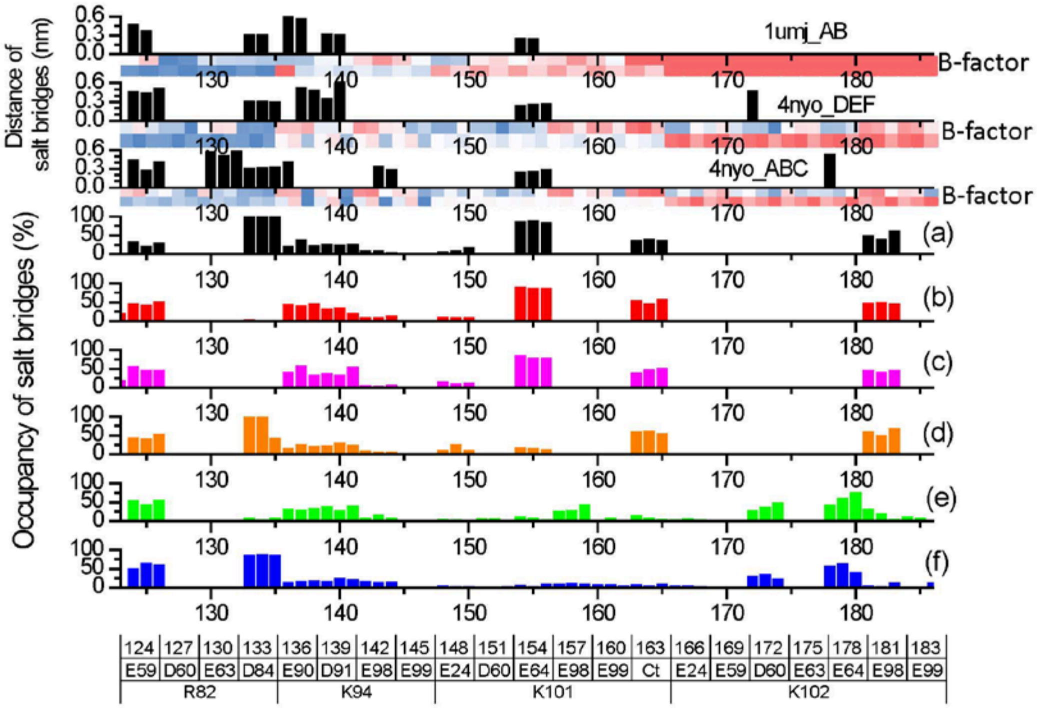

Fig. S3B

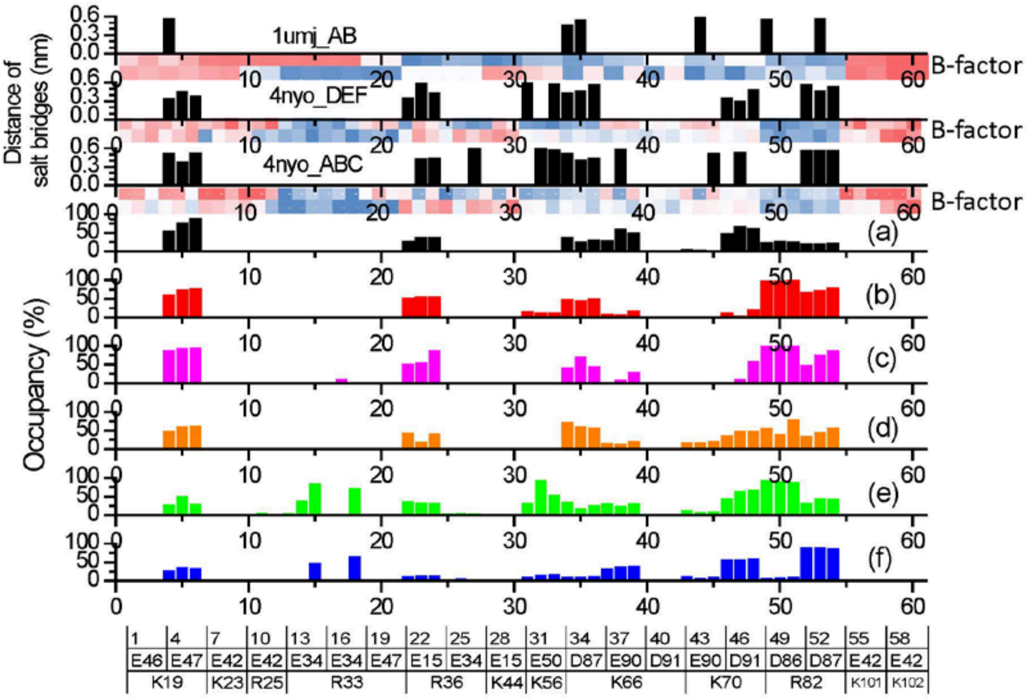

Figure S4. Snapshot of the configuration around Arg68 of PhCutA1

Green and cyan represent snapshots at 100 ns of 300 K MD simulations for Amber99sb_tip3p and Amber14sb_tip3p, respectively.

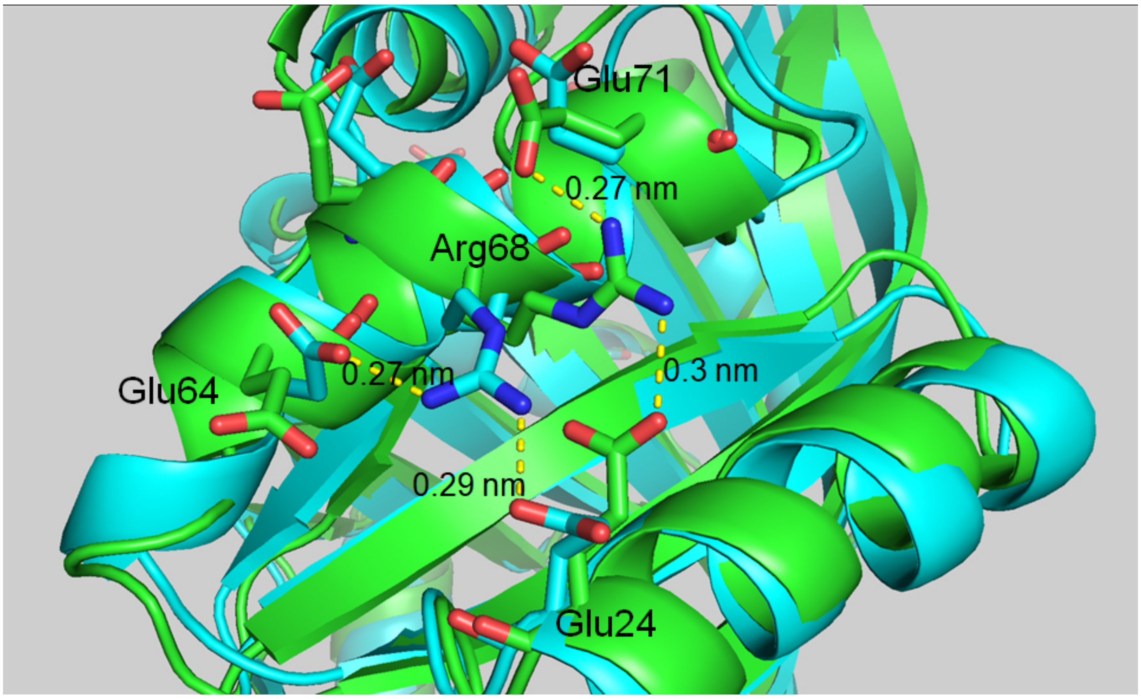

Figure S5. The configuration around Arg82 of PhCutA1.

Green, cyan, and magenta represent A, B, and C-subunits of PhCutA1, respectively.

(A) The crystal structure of PhCutA1 (A, B, and C-subunits of 4nyo).

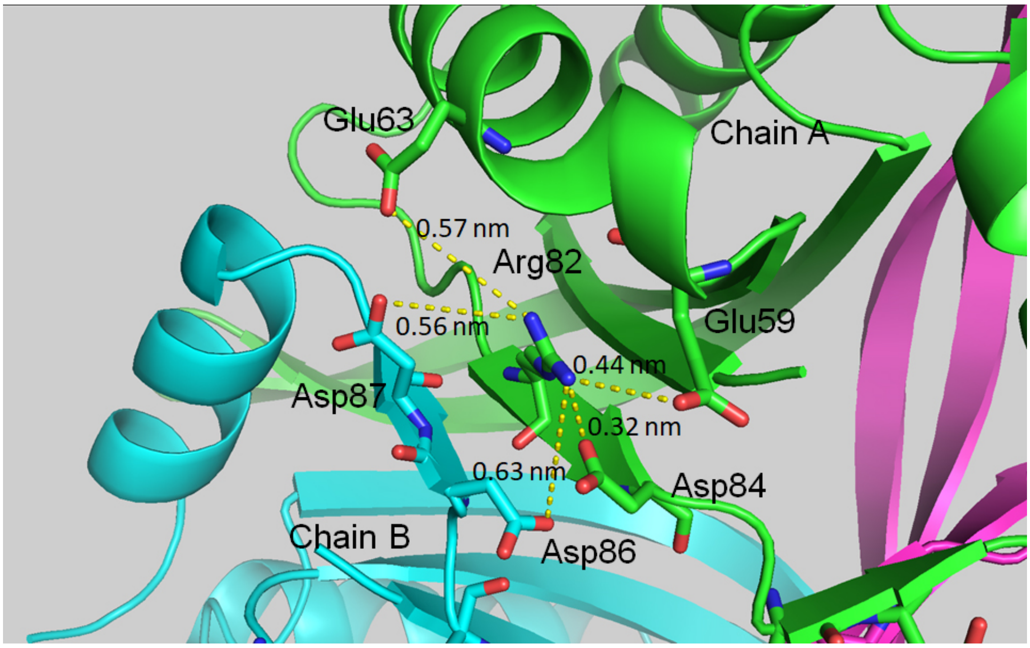

(B) The snapshot around Arg82 of PhCutA1 at 200 ns of an MD simulation in the case of Gromos53a6_spc/e.

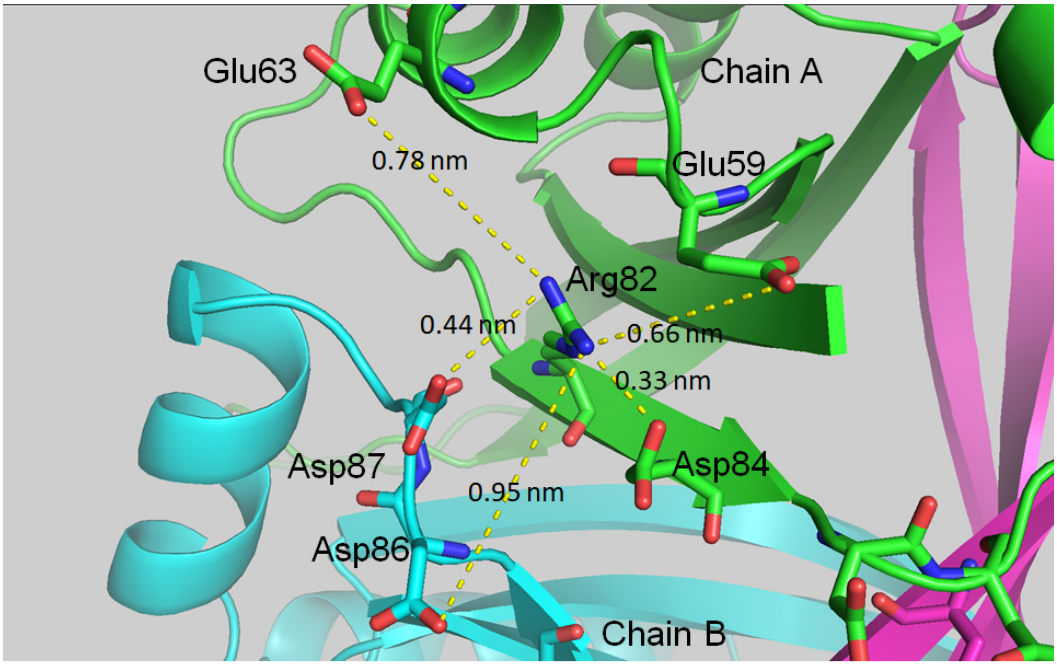

Figure S6. The snapshot of the configuration around N-terminal (Met1) of PhCutA1 at 100 ns of an MD simulation using Amber99sb_tip3p.

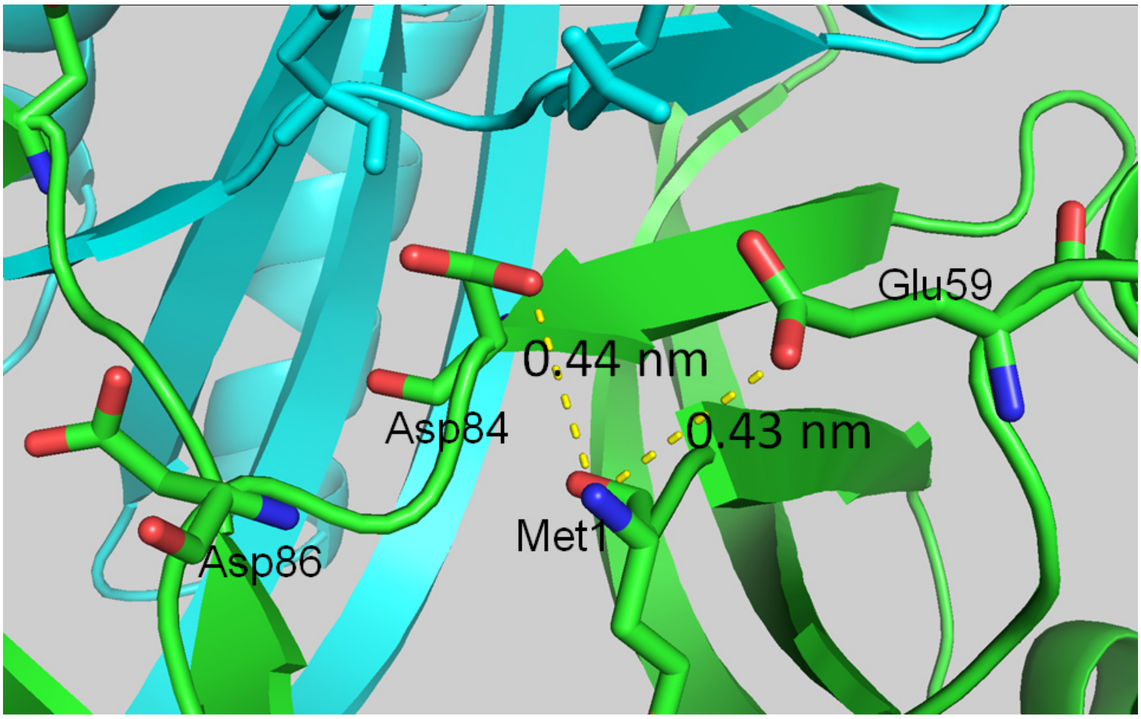

Figure S7. The snapshots of the configuration around Lys44 of PhCutA1 at 100 ns in an MD simulation using Charmm27_tip3p (A) and Gromos53a6_spc/e (B)

(S7A)

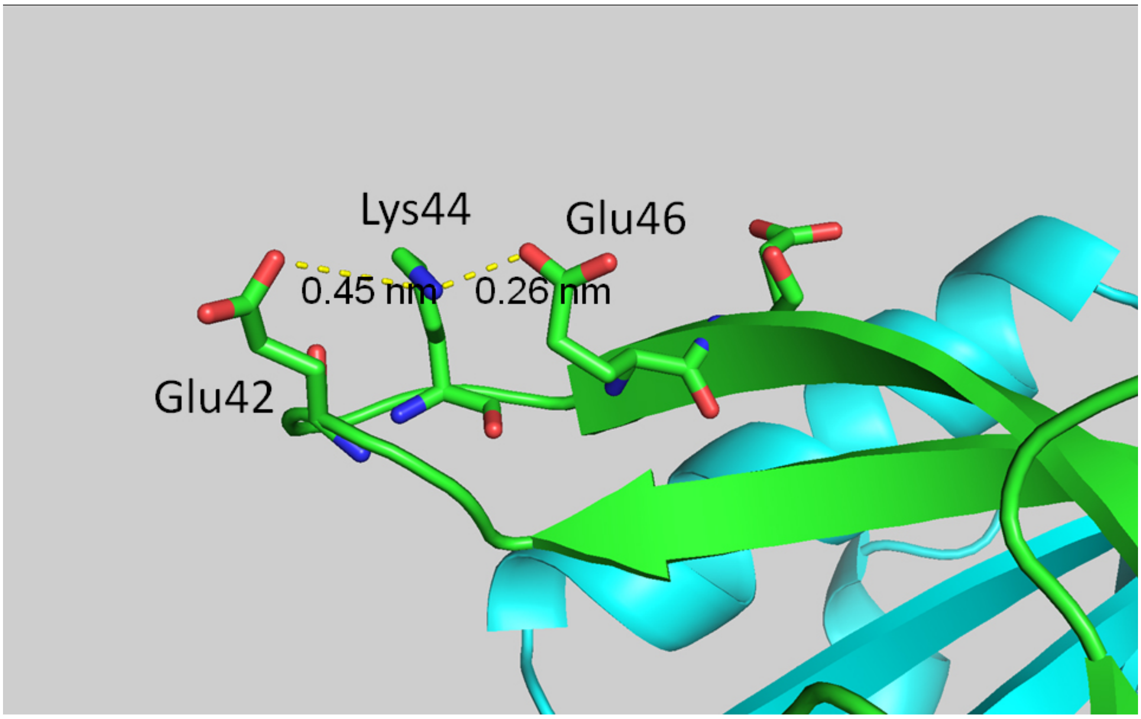

(S7B)

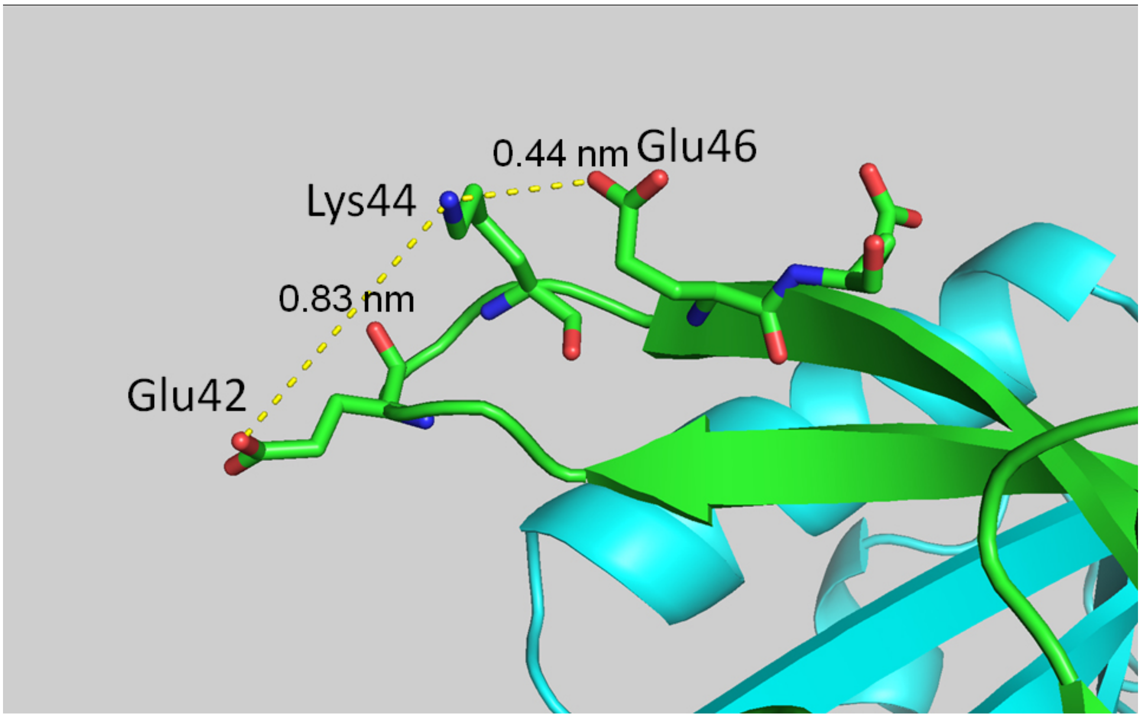

Figure S8. The snapshots of the configuration around Lys70 of PhCutA1 at 100 ns in an MD simulation using Gromos43a1_spc/e (A) and Amber99sb_tip3p (B)

(S8A)

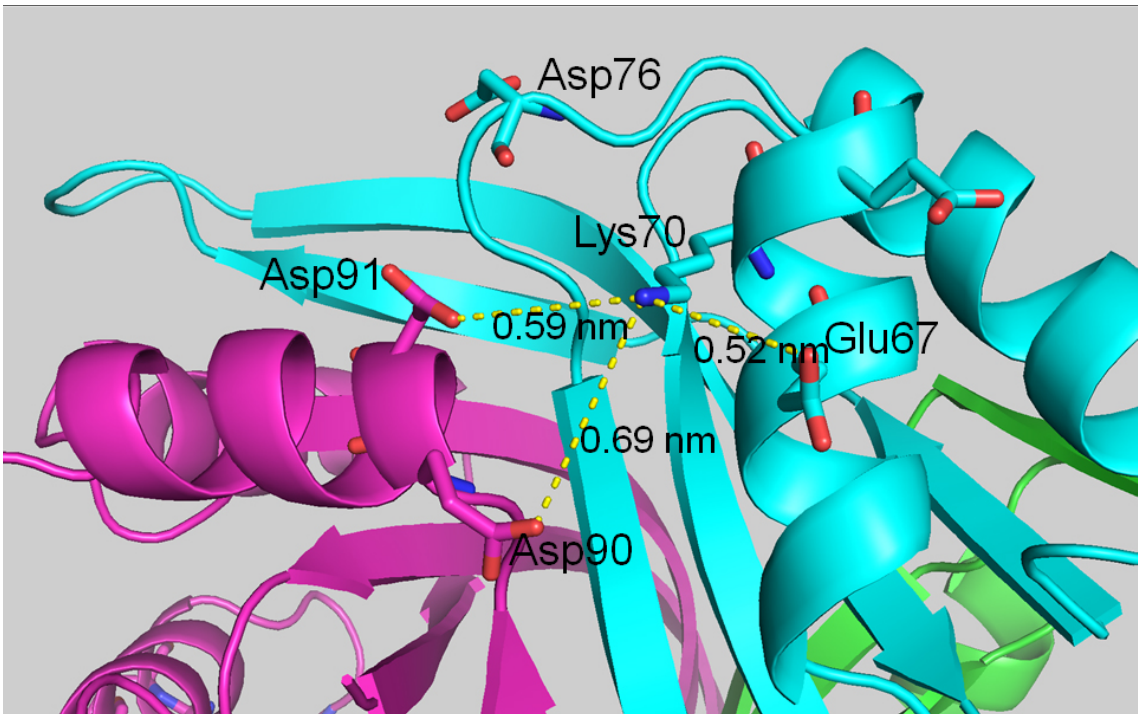

(S8B)

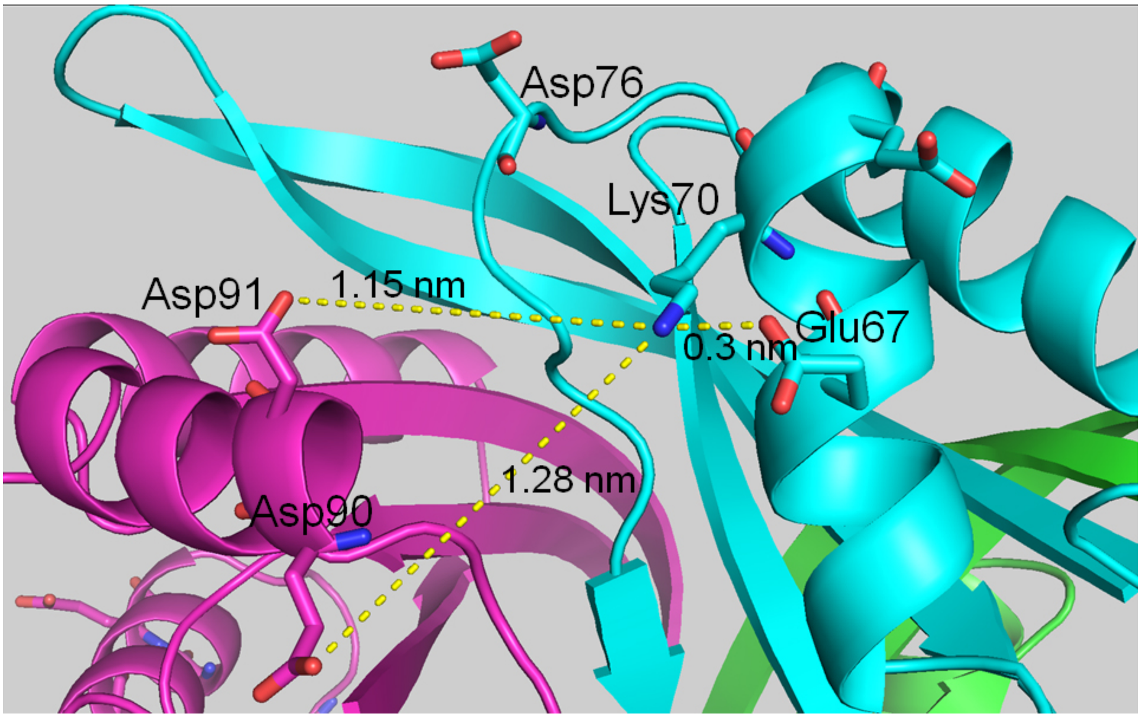

Figure S9. The snapshots of the configuration around Arg36 of PhCutA1 at 200 ns in an MD simulation using Amber99sb_tip3p (A) and Gromos53a6_spc/e (B)

(S9A)

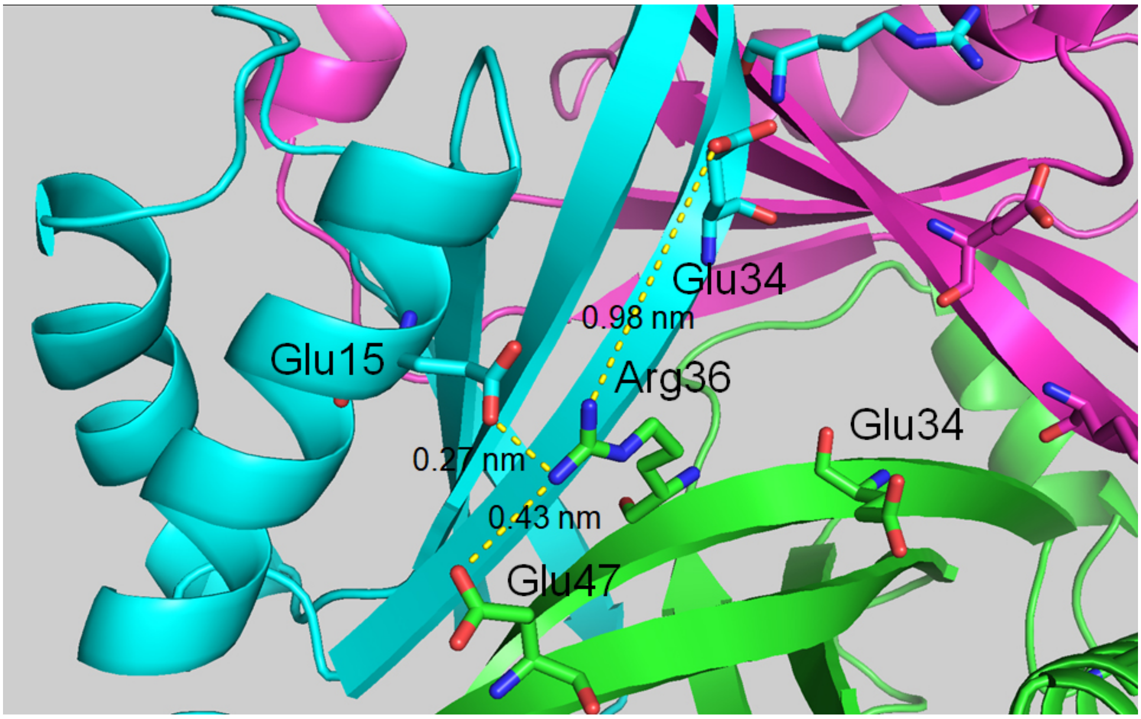

(S9B)

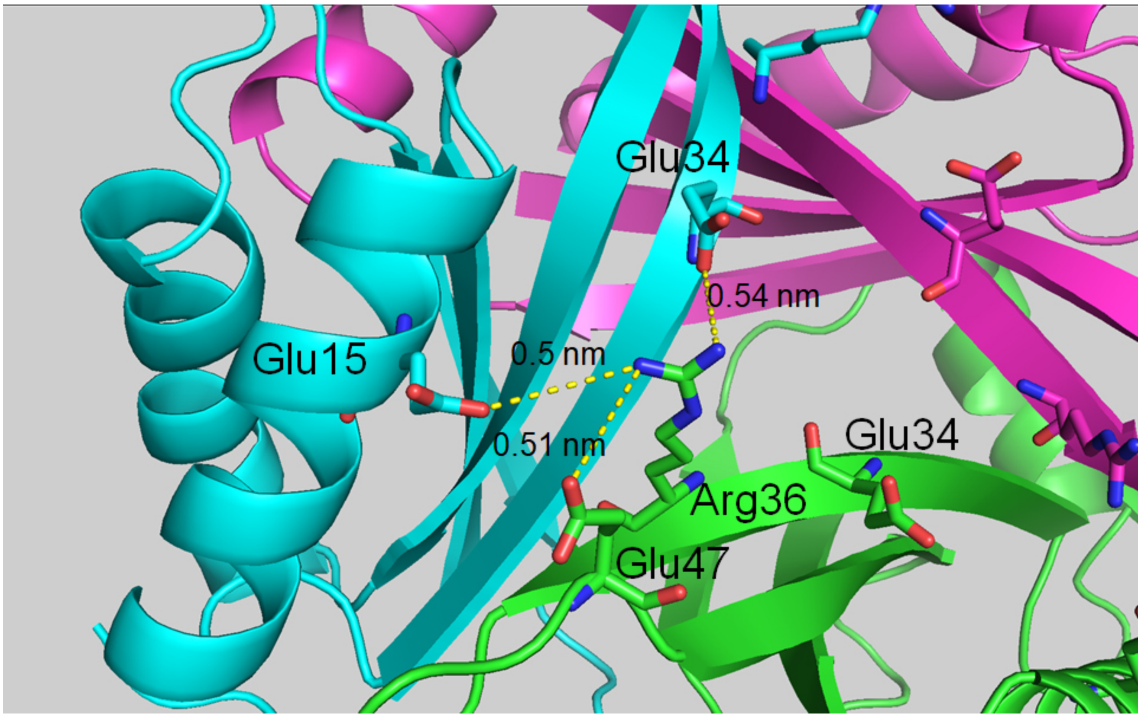

Figure S10. The snapshots of the configuration around Lys101 and Lys102 of PhCutA1 at 100 ns in an MD simulation using Charmm27_tip3p (A) and Gromos53a6_spc/e (B)

(S10A)

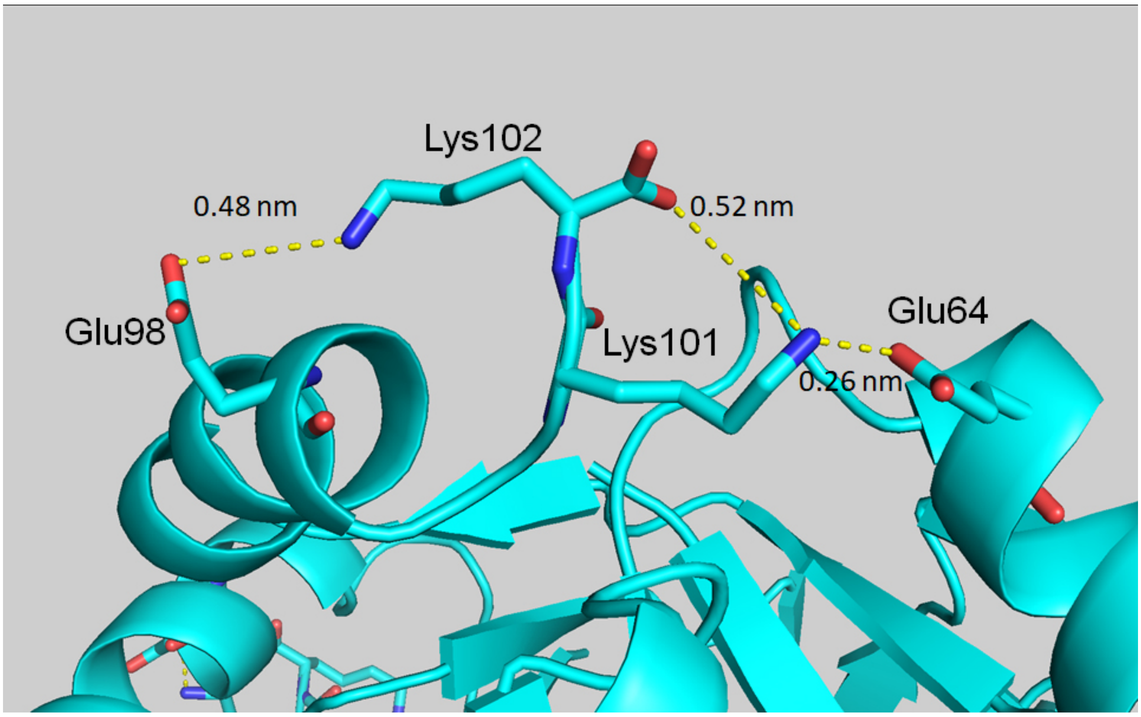

(S10B)

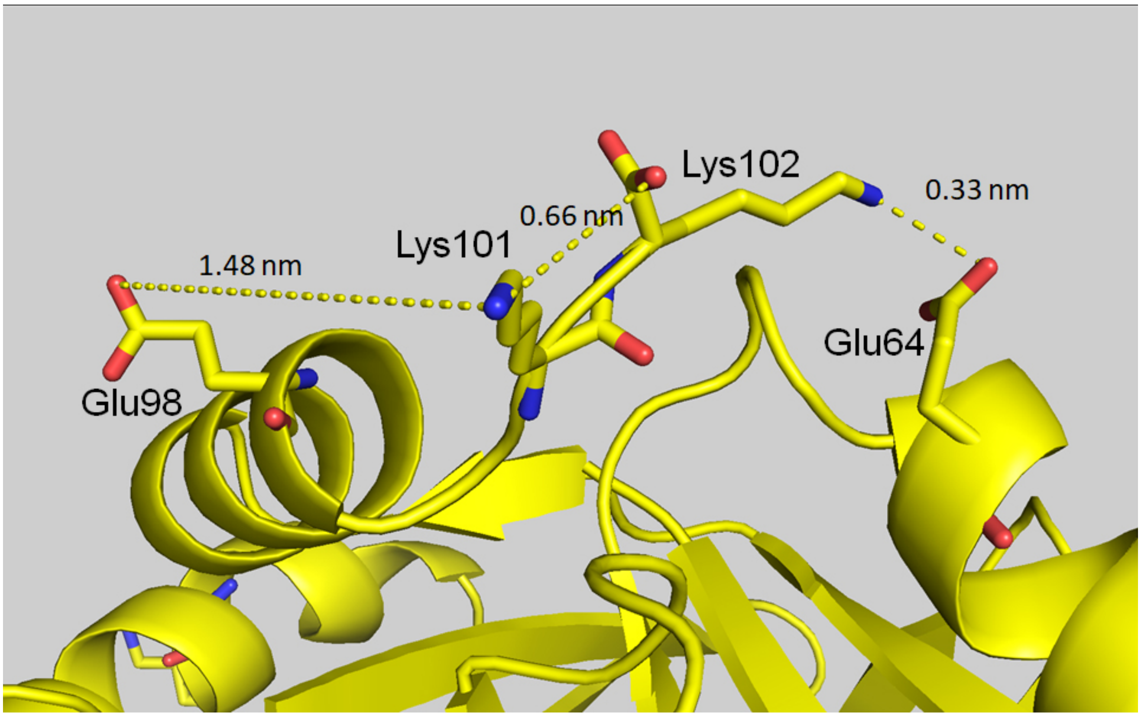

Figure S11. The snapshots of the configuration around Arg58 of PhCutA1 at 200 ns in an MD simulation using Amber14_tip3p (A) and Gromos43a1_spc/e (B)

(S11A)

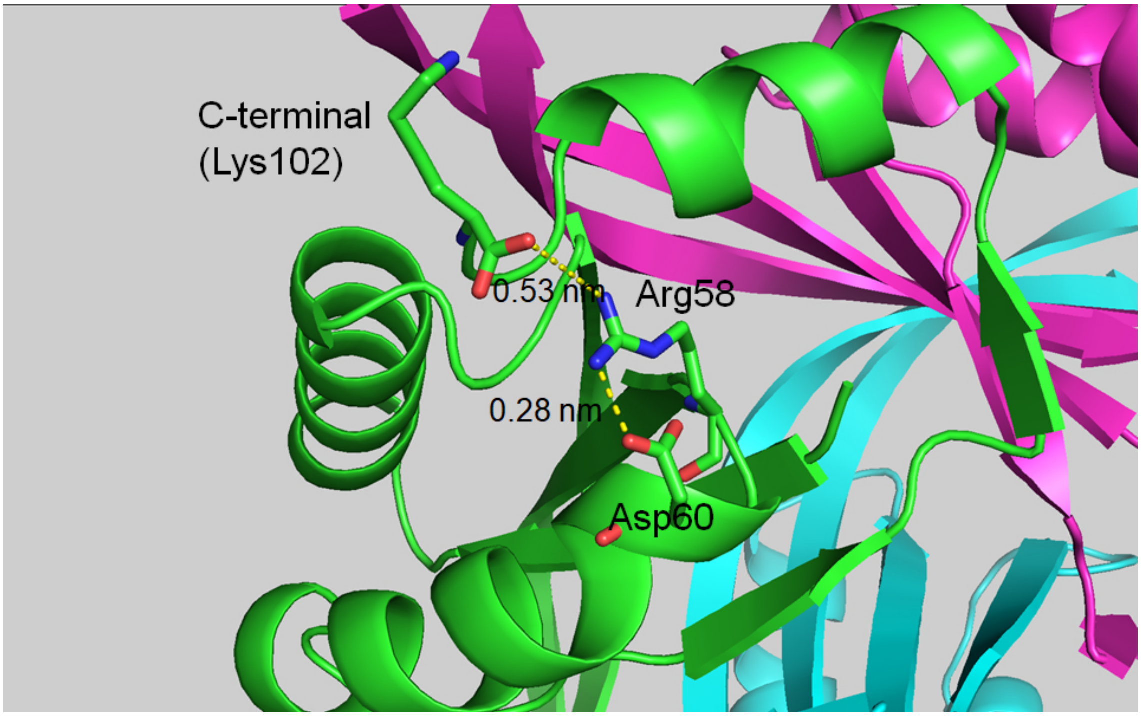

(S11B)

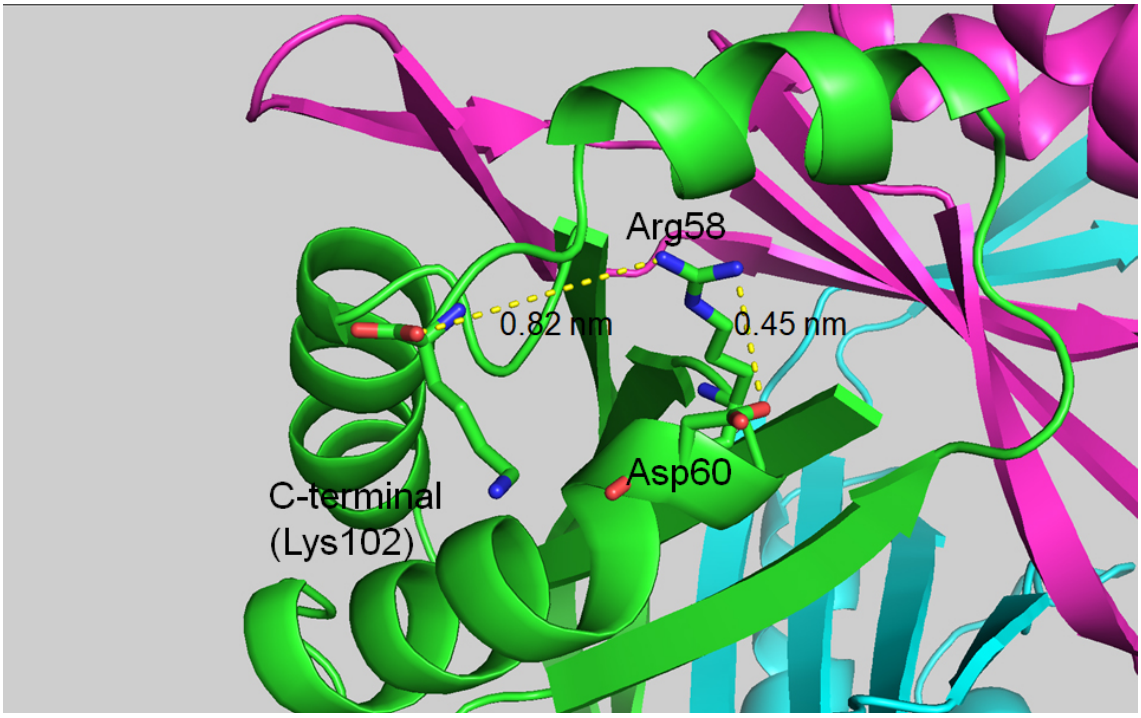

Figure S12. The snapshots of the configuration around Glu50 interacting with Lys56 of PhCutA1 at 100 ns in an MD simulation using Gromos43a1_spc/e (A) and Amber99sb_tip3p (B)

Green, cyan, and magenta represent A, B, and C-subunits of PhCutA1, respectively.

(S12A)

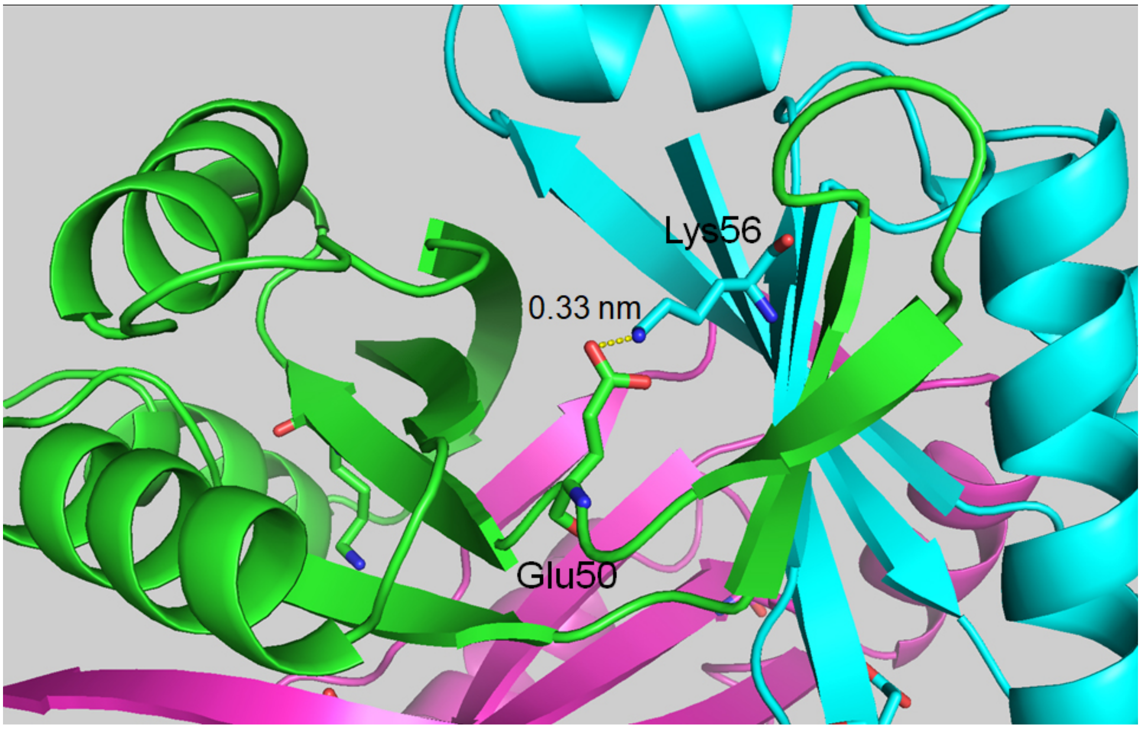

(S12B)

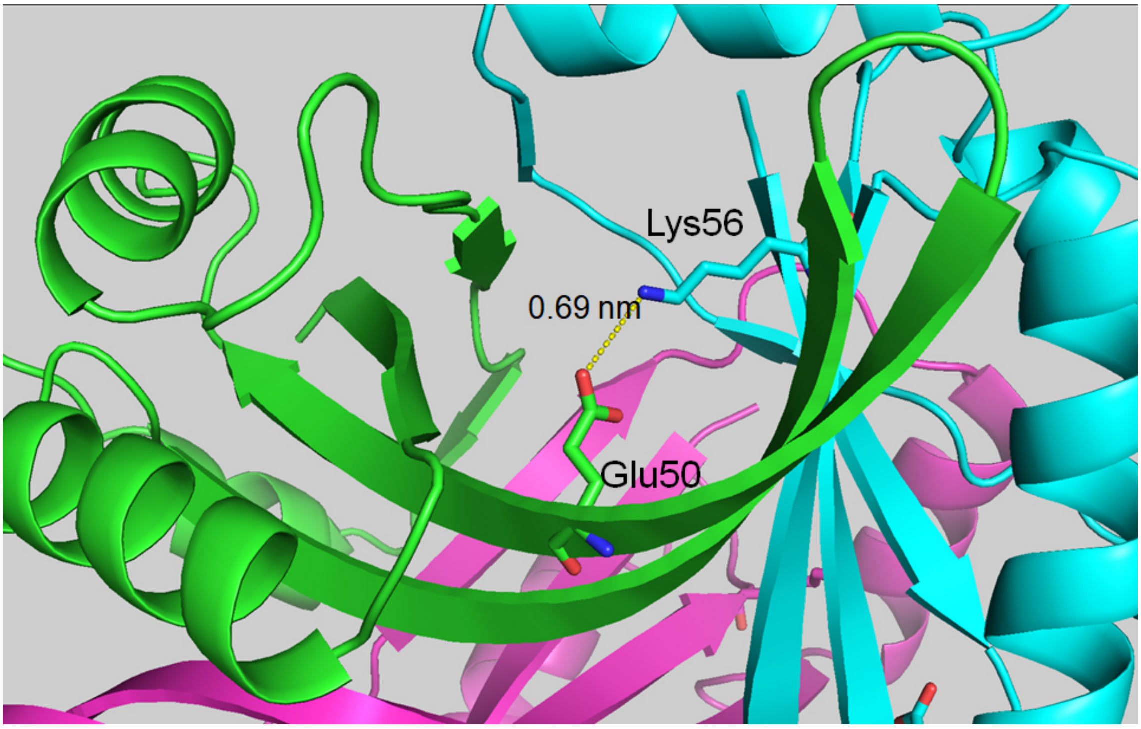

Figure S13. The configuration around Asp84 and Asp86 of PhCutA1.

Green, cyan, and magenta represent A, B, and C-subunits of PhCutA1, respectively.

(A) The crystal structure of PhCutA1 (A, B, and C-subunits of 4nyo).

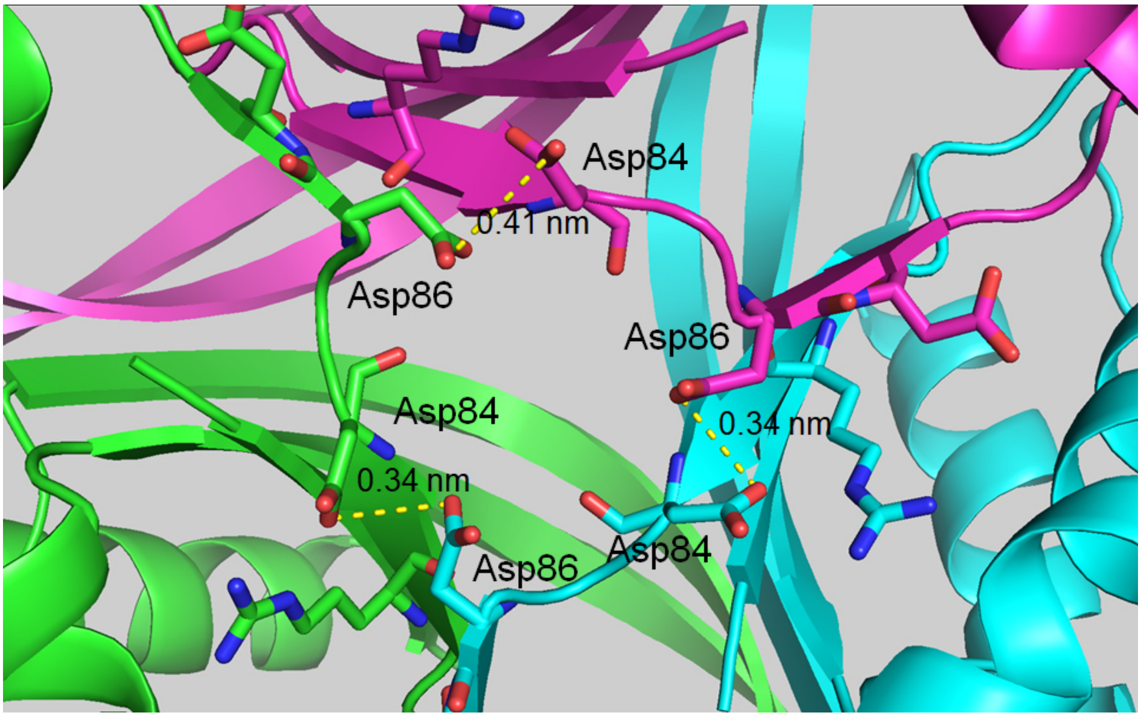

(B) The snapshot of PhCutA1 at 200 ns of an MD simulation in the case of Gromos43a1_spc/e.

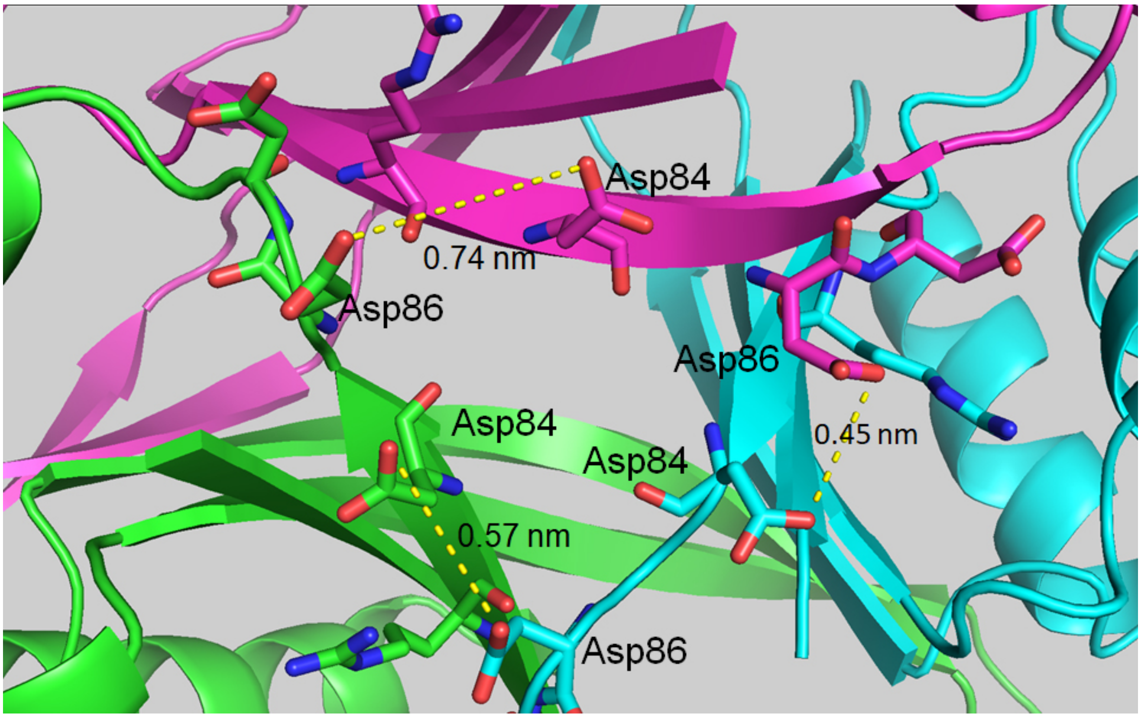

Figure S14. The configuration around Arg33 of PhCutA1

(A) Snapshots around Arg33 and Glu34 of PhCutA1 at 100 ns of an MD simulation using Gromos43a1_spc/e. Green, cyan, and magenta represent A, B, and C-subunits of PhCutA1, respectively.

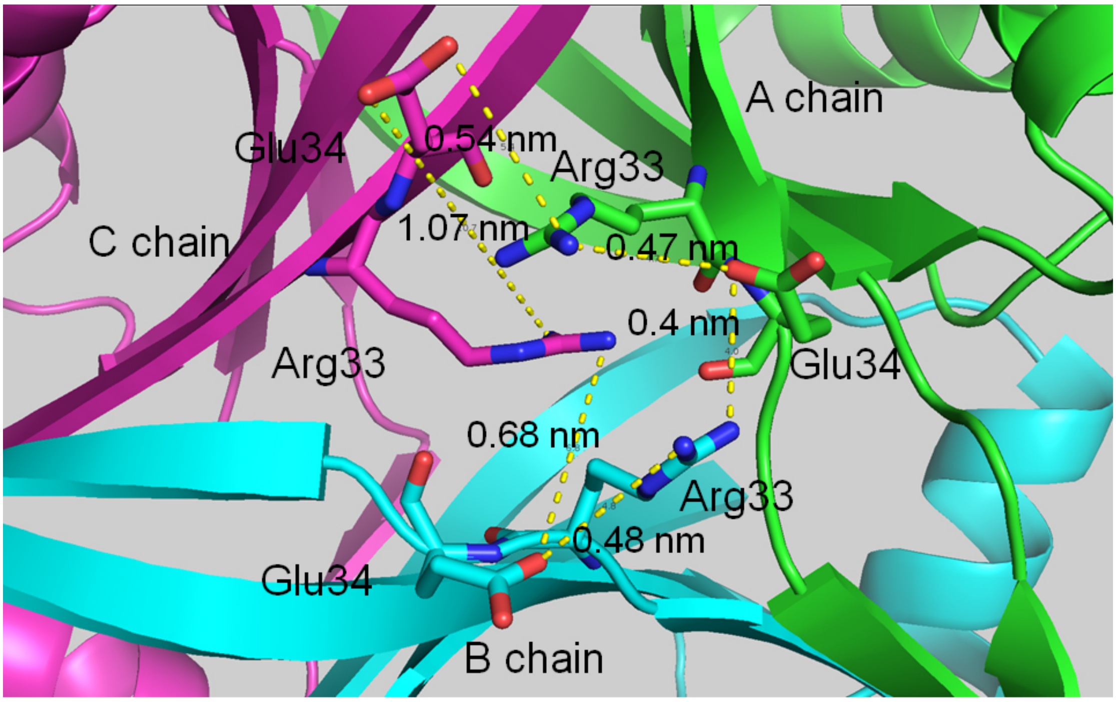

(B) Snapshots around Arg33 of PhCutA1 at 100 ns of MD simulation in the cases of Gromos43a1_spc/e (cyan) and Amber99sb_tip3p (yellow).

Both structures are superimposed.

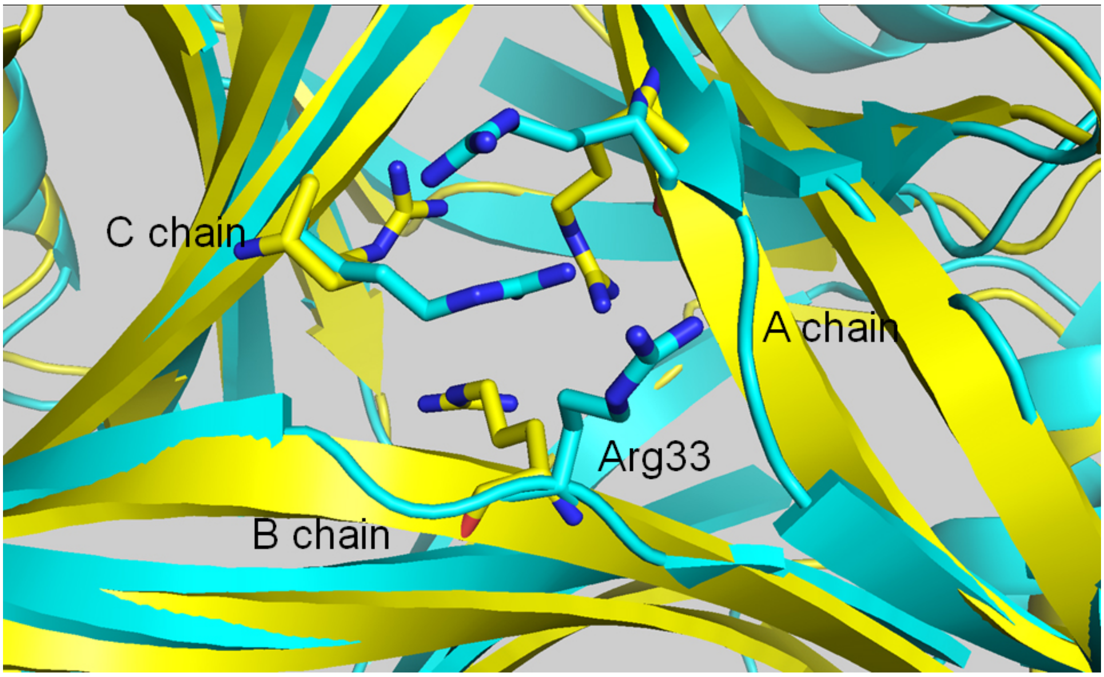

Figure S15. Trajectories of distance between Arg33 in PhCutA1 and Cl^- ion during 400-ns MD simulations at 300 K using indicated force fields

(a) Charmm27_tip3p, the distance between C_ζ of Arg33 in A-subunit and Cl^- ion of the number 12197. The percent occupancy of distance (less than 0.6 nm) between them was 100.0 %.

(b) Amber99sb_tip3p, the distance between C_ζ of Arg33 in A-subunit and Cl^- ion of the number 12197. The percent occupancy of distance (less than 0.6 nm) between them was 86.0 %.

(c) Gromos43a1_spc/e, the distance between C_ζ of Arg33 in C-subunit and Cl^- ion of the number 12220. The percent occupancy of distance (less than 0.6 nm) between them was 1.7 %.

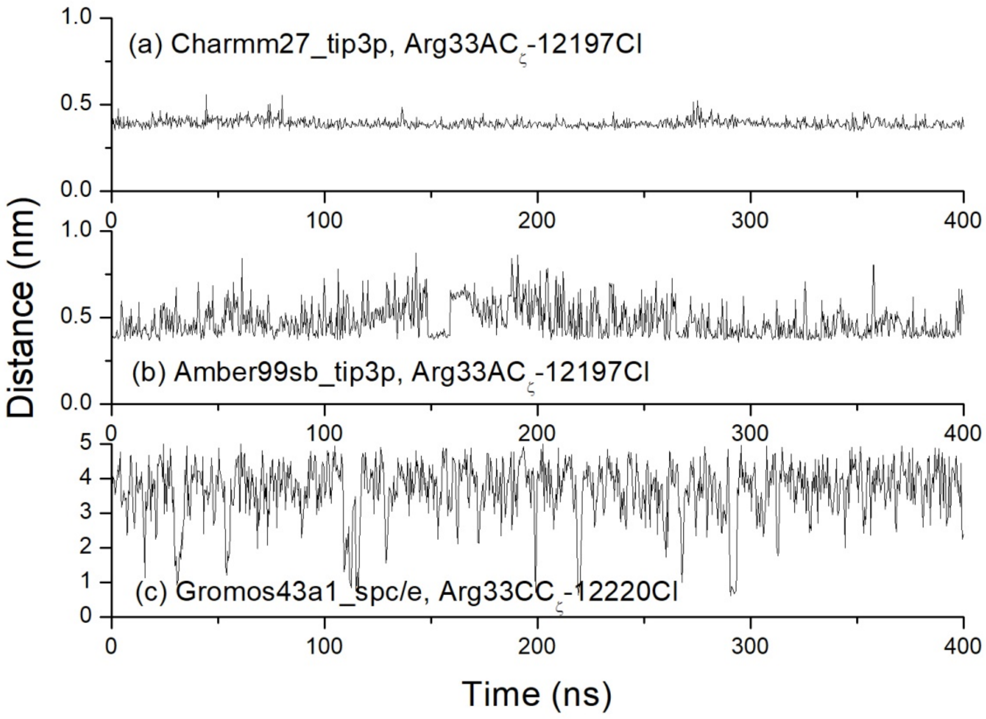

Supplement: Supplementary file 1 — Table S1 A, 186 intra‐subunit interactions between favorable ion pairs in PhCutA1. B, 60 inter‐subunit interactions between favorable ion pairs in PhCutA1. Table S2. Number of residues of PhCutA1 in each type of secondary structure in MD simulations (50–400 ns). Table S3 A, Average distance between favorable intra‐subunit salt bridges in PhCutA1 during 400‐ns MD simulations using the indicated force fields. B, Average distance between favorable inter‐subunit salt bridges in PhCutA1 during 400‐ns MD simulations using the indicated force fields. Table S4. Electrostatic energy of targeted residues for two structures from crystal analysis and six structures from MD simulation of PhCutA1. Table S5. Comparison of percent occupancy of intra‐subunit salt bridges in PhCutA1 at each 100 ns during 400 ns MD simulation at 300 K using indicated force fields. Table S6. Comparison of percent occupancy of intra‐subunit salt bridges in each subunit of PhCutA1 during 400 ns MD simulation at 300 K using indicated force fields. Table S7. Side‐chain rotamer criteria of charged residues in PhCutA1. Table S8. Buried ratio and pKa of negatively and positively charged residues in the crystal structure of PhCutA1. Table S9. Buried ratio and pKa of negatively and positively charged residues in the crystal structure of PhCutA1. Table S10. The number of Na+, Cl−, and H2O in the simulation box and size of the box during MD simulations at 300 K using indicated force fields. Fig. S1. Comparison of helicity for PhCutA1 among six force fields in 300 K MD simulations (50–400 ns). (A) Percent helicity shows average values for each residue of PhCutA1. Red, Blue, and Black represent Charmm27_tip3p, Amber99sb_tip3p, and others, respectively. (B) Difference in helicity (Helicity subtraction of Amber99sb_tip3p from Charmm27_tip3p). Fig. S2. Comparison of root‐mean‐square fluctuation (RMSF) for the C⍺ atoms of PhCutA1 among six force fields in 300 K MD simulations (50–400 ns). (A)Average RMSF values at each [file FEB4-9-1939-s001.pdf]
